# Interleukin-4 and interleukin-13 increase NADPH oxidase 1-related proliferation of human colon cancer cells

**DOI:** 10.18632/oncotarget.17494

**Published:** 2017-04-27

**Authors:** Han Liu, Smitha Antony, Krishnendu Roy, Agnes Juhasz, Yongzhong Wu, Jiamo Lu, Jennifer L. Meitzler, Guojian Jiang, Eric Polley, James H. Doroshow

**Affiliations:** ^1^ Division of Cancer Treatment and Diagnosis, National Cancer Institute, Bethesda, Maryland, USA; ^2^ The Center for Cancer Research, National Cancer Institute, Bethesda, Maryland, USA

**Keywords:** NADPH oxidase, reactive oxygen species, colon cancer, interleukin-4, interleukin-13

## Abstract

Human colon cancers express higher levels of NADPH oxidase 1 [NOX1] than adjacent normal epithelium. It has been suggested that reactive oxygen species [ROS] derived from NOX1 contribute to DNA damage and neoplastic transformation in the colon, particularly during chronic inflammatory stress. However, the mechanism(s) underlying increased NOX1 expression in malignant tumors or chronic inflammatory states involving the intestine are poorly characterized. We examined the effects of two pro-inflammatory cytokines, IL-4 and IL-13, on the regulation of NOX1. NOX1 expression was increased 4- to 5-fold in a time- and concentration-dependent manner by both cytokines in human colon cancer cell lines when a functional Type II IL-4 receptor was present. Increased NOX1 transcription following IL-4/IL-13 exposure was mediated by JAK1/STAT6 signaling, was associated with a ROS-related inhibition of protein tyrosine phosphatase activity, and was dependent upon activation and specific binding of GATA3 to the NOX1 promoter. NOX1-mediated ROS production increased cell cycle progression through S-phase leading to a significant increase in cellular proliferation. Evaluation of twenty pairs of surgically-resected colon cancers and their associated uninvolved adjacent colonic epithelium demonstrated a significant increase in the active form of NOX1, NOX1-L, in tumors compared to normal tissues, and a significant correlation between the expression levels of NOX1 and the Type II IL-4 receptor in tumor and the uninvolved colon. These studies imply that NOX1 expression, mediated by IL-4/IL-13, could contribute to an oxidant milieu capable of supporting the initiation or progression of colonic cancer, suggesting a role for NOX1 as a therapeutic target.

## INTRODUCTION

Although a robust flux of reactive oxygen species [ROS] may produce significant tissue injury [1], low intracellular ROS levels, especially of H_2_O_2_, can play a critical role in signal transduction [2, 3], providing essential proliferative signals required for tumor cell growth [4] and angiogenesis [5, 6]. ROS are also strongly associated with pro-inflammatory states that contribute significantly to carcinogenesis and tumor progression [7, 8]. Our understanding of the mechanisms of ROS formation in tumors has been enhanced during the past decade by the discovery of six NADPH oxidases [NOX1,3,4, and 5; DUOX1 and 2] in mammalian cells that are structural homologues of gp91^phox^ [NOX2], the major membrane-bound component of the respiratory burst oxidase of leukocytes [9–11].

The first of the NOX2 homologues to be described, NOX1, was discovered through study of Caco2 human colon cancer cells [12]; recent experiments have clarified the role of NOX1-mediated ROS production in colon cancer cell migration, integrin signaling, proliferation, and carcinogenesis [13–17]. In addition, while NOX1 appears to contribute to gastrointestinal host defense and wound healing [18–20], there is also evidence that pre-malignant, chronic inflammation of the colon (in mouse models) is associated with functional expression of NOX1 [21]; hence, NOX1 could contribute to the pathogenesis of inflammation-related colonic malignancies. These recent genetic studies are consistent with the demonstration of enhanced NOX1 expression *in vitro* following exposure of intestinal cancer cells to the pro-inflammatory cytokines interferon-γ [IFN-γ] and tumor necrosis factor-α [TNF-α] [22]. Despite the fact that a wide range of inflammatory cytokines has been associated with pre-malignant chronic inflammation of the colon and inflammatory bowel disease [23], gaps exist in our understanding of the regulatory mechanisms (beyond plasma membrane association or phosphorylation of components of the NOX1 complex) [24, 25] that control NOX1 expression in the colon, particularly in response to inflammatory stimuli.

Our laboratory recently demonstrated that small molecule inhibitors of NOX1 decrease human colon cancer cell proliferation both *in vitro* and in human tumor xenografts [17]. Using a bioinformatics approach, we found that the pattern of NOX1 inhibitor-related growth delay across a large human tumor cell line panel (the NCI-60) was significantly related to the expression of inflammation-related genes, including the cytokine interleukin-4 [IL-4] and components of the JAK/STAT pathway [26]. In support of this hypothesis, we demonstrated that exposure of human colorectal cancer cells to clinically-achievable concentrations of the NOX (and related flavin dehydrogenase) inhibitors diphenylene iodonium [DPI] or 2-di-thienyl-iodonium [DTI], which decreased intracellular ROS levels, blocked IL-4- and IL-13-induced phosphorylation of STAT1, 3, and 6, as well as signaling through the mitogen activated protein kinase [MAPK] pathway. These experiments suggested that ROS generated by NOX1 might affect IL-4/IL-13-dependent signal transduction events in colon cancer.

IL-4 and IL-13, produced by activated T helper type 2 [T_H_2] lymphocytes and other immune cells, were discovered over 25 years ago [27]; the focus of most investigation since that time has been on the important roles of these cytokines in immuno-surveillance [28], the induction of immunoglobulin switching in B cells and the pathology of asthma [29], as well as macrophage polarization. Recent studies, however, have also emphasized the growth-promoting and pro-metastatic roles of these cytokines that are often highly expressed intracellularly, as well as in the surrounding microenvironment, in a wide variety of epithelial cancers, including colorectal cancer [30–37]. Binding of IL-4 or IL-13 to the Type II IL-4 receptor [IL-4Rα], which is found on non-lymphoid cells, initiates a signaling cascade that activates the JAK/STAT pathway (particularly STAT6) as well as MAPK and Akt cell-survival functions; one biochemical consequence of receptor activation is a context-dependent increase in the expression of anti-apoptotic proteins that can contribute to enhanced cell proliferation and resistance to cancer therapy [38, 39]. IL-13 may also signal through AP-1-dependent pathways (and the separate IL-13Rα2), independent of those pathways activated by IL-4, to increase invasion and metastasis [40].

A relationship between reactive oxygen production and IL-4 function was postulated by Sharma and colleagues [41] who suggested that exposure of the A549 human lung adenocarcinoma cell line to IL-4 activated NOX1 to generate ROS within minutes, without changing NOX1 expression levels; they suggested that subsequent, ROS-related inhibition of protein tyrosine phosphatase activity could play an important, enhancing role in IL-4 signaling.

In contrast, the experiments reported herein demonstrate that human colon cancer cell lines significantly increase NOX1 expression (but not that of other NOXs) following 12 to 120 h of continuous exposure to IL-4 or IL-13; ROS production in these cells is associated with the presence of NOX1 protein at the plasma membrane surface. Furthermore, NOX1-dependent ROS enhance colon cancer proliferation by increasing cell cycle progression through S phase. Increased NOX1 transcription is directly related to activation of the JAK1/STAT6 pathway and is mediated by the binding of the GATA3 transcription factor to the NOX1 promoter. Finally, the clinical relevance of these *in vitro* experiments is supported by our demonstration that the expression of NOX1 at the mRNA level in human colon cancers is significantly correlated with the expression of IL-4Rα, suggesting that IL-4-dependent NOX1 expression may be a novel therapeutic target in this disease.

## RESULTS

### NOX1 expression in human colon cancer cells is increased by IL-4 or IL-13 treatment

**Figure 1:**
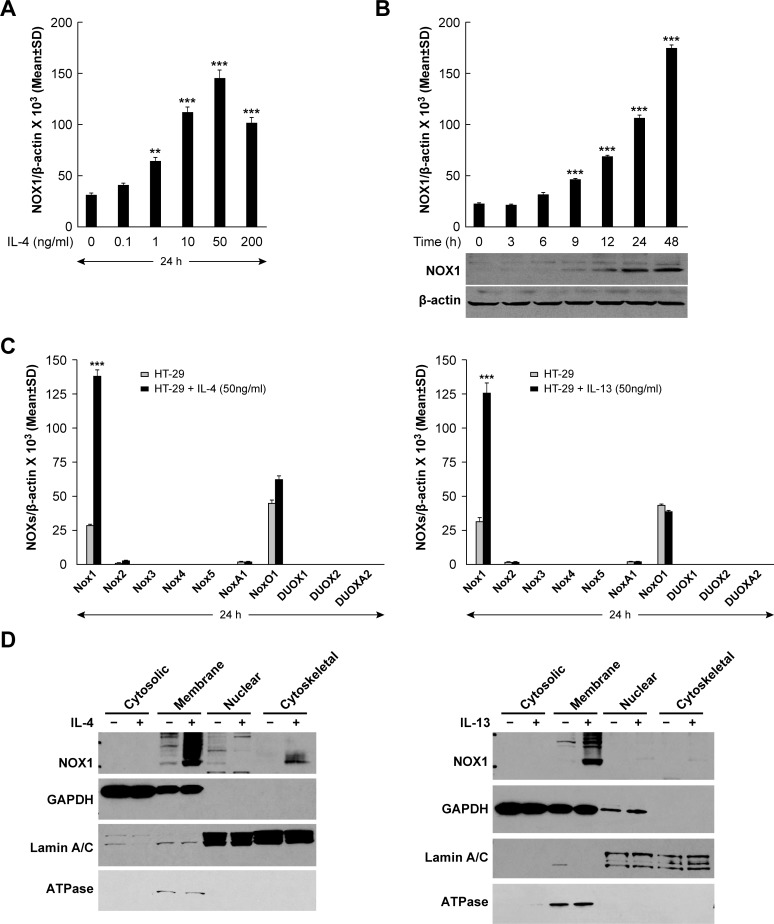
Expression of NOX1 in HT-29 cells following IL-4/IL-13 treatment **A**. IL-4 induced a dose-dependent increase in NOX1 expression; HT-29 cells were treated with IL-4 at different concentrations (0, 0.1, 1, 10, 50, 200 ng/ml) for 24 h. NOX1 levels were analyzed by quantitative RT-PCR with β-actin serving as the internal control. **B**. HT-29 cells were treated with IL-4 (50 ng/ml) for different lengths of time (0, 3, 6, 9, 12, 24, and 48 h); levels of NOX1 expression were determined using quantitative RT-PCR and Western analysis with β-actin serving as the internal control. **C**. IL-4 and IL-13 specifically induced the expression of NOX1 but not of other NOX family members. HT-29 cells were treated with IL-4 (left) or IL-13 (right) at a concentration of 50 ng/ml for 24 h. Quantitative RT-PCR was performed to evaluate the expression levels of the individual NOX family members and their accessory genes; expression levels were normalized to β-actin. **D**. NOX1 protein induced by exposure to IL-4 or IL-13 is localized to the plasma membrane. HT-29 cells were treated with IL-4 (left) or IL-13 (right) at a concentration of 50 ng/ml for 24 h. Western analysis was performed on subcellular fractions (cytosolic, membrane, nuclear and cytoskeletal) to determine the localization of NOX1. GAPDH, ATPase, and Lamin A/C were used as cytosolic, membrane, and nuclear markers, respectively. Data represented as mean ± SD values; ** = *P* < 0.01; *** = *P* < 0.001.

To examine the relationship between exogenous IL-4 or IL-13 exposure and the expression of NOX1, we first determined whether the NOX1-expressing colon cancer cell lines under study possessed IL-4Rα, as has been reported previously [31]. We confirmed by real time RT-PCR that IL-4Rα mRNA is present in all of the human colon cancer cells utilized (Supplementary Figure S1A). When HT-29 cells were treated with IL-4, NOX1 mRNA expression increased in a concentration- and time-dependent manner, as early as 12 h following cytokine exposure, and at IL-4 concentrations as low as 1 ng/ml (*P* < 0.01) (Figures 1A and 1B). Because NOX1 levels were maximal following exposure to 50 ng/ml of IL-4 (≈ 4 to 5-fold increase), that concentration was employed in subsequent experiments. Western analysis revealed that increased NOX1 protein levels correlated with the increase in NOX1 mRNA produced by IL-4 exposure (Figure 1B). Furthermore, expression level changes following IL-4 treatment were specific for NOX1; none of the other members of the NOX family, nor the accessory genes associated with NOX activity, demonstrated a similar pattern of response following the addition of IL-4 to HT-29 cells (Figure 1C). In concert with these results, we found that IL-4 increased NOX1 expression in other colon cancer lines, including DLD-1, WiDr, NCI-H508, and SW403 cells (Supplementary Figure S1B). Because IL-4 and IL-13 are functionally related and share the Type II IL-4R [42, 43], we also evaluated the response of HT-29 cells to IL-13; as expected, IL-13 specifically increased the expression of NOX1 (Figure 1C). Furthermore, we found that the expression of NOX1 was also increased by IL-13 in DLD-1, WiDr, NCI-H508, and SW403 cells (Supplementary Figure S1B). Because functional NOX1 protein exists as a multi-component plasma membrane complex [44], subcellular fractionation studies were performed to determine the localization of NOX1 protein following IL-4 or IL-13 exposure. Cytokine-enhanced NOX1 protein expression co-purified with the plasma membrane marker Na^+^/K^+^ ATPase, demonstrating the plasma membrane localization of the NOX1 that had been expressed (Figure 1D). Similar experiments utilizing IL-4-treated DLD-1 colon cancer cells confirmed the plasma membrane location of the NOX1 that had been produced (Supplementary Figure S1C). We also found for DLD-1 cells that the effect of IL-4 exposure on NOX1 expression was time-dependent (Supplementary Figure S1D).

### Increased NOX1 expression and ROS production following cytokine exposure significantly enhance the proliferation of colon cancer cells

**Figure 2:**
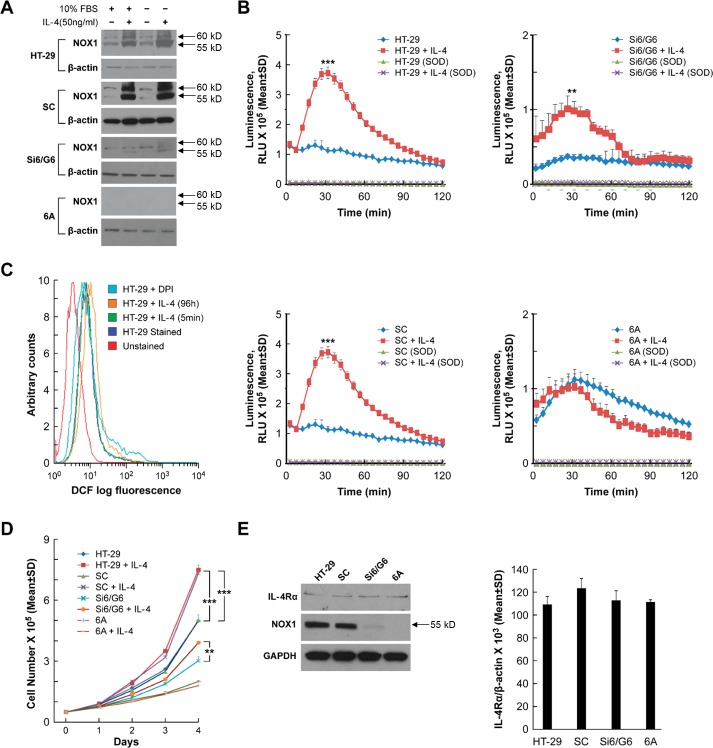
Effect of IL-4 on NOX1 protein expression, ROS production, and cell proliferation in HT-29 cells **A**. The effect of IL-4 on the protein expression of NOX1 is demonstrated by Western analysis under both serum-free conditions and in the presence of 10% FBS for both parental HT-29 cells and a clonal line selected for expression of a scrambled shRNA (SC cells). Modest to no effect of IL-4 on NOX1 expression is shown for the Si6/G6 intermediate NOX1 knockdown clones, or for 6A cells (> 90% NOX1 knockdown), respectively. **B**. NOX1 protein levels correlate with relative superoxide production following IL-4 exposure. Compared to superoxide production in HT-29 and SC cells exposed to IL-4, superoxide levels were only modestly increased in Si6/G6 cells, and were not changed at all by IL-4 treatment in 6A cells. **C**. IL-4 treatment increases ROS production in HT-29 cells. HT-29 cells were treated with IL-4 for the indicated times, harvested, and stained with CM-H_2_-DCFDA. Flow cytometry was performed to evaluate intracellular ROS levels. Compared with parental HT-29 cells, exposure to IL-4 for 96 h shifted intracellular fluorescence intensity to the right, indicating higher ROS levels; treatment with IL-4 for 5 min, however, produced no shift in DCF fluorescence. Pretreatment with the flavin dehydrogenase and NOX inhibitor DPI produced a left shift in fluorescence intensity. **D**. IL-4 (50 ng/ml) promotes HT-29 and SC cell proliferation. Cell numbers were counted daily in the presence or absence of IL-4 for 4 days. After the 4-day IL-4 treatment, compared with corresponding controls, HT-29 and SA cells showed a nearly 2-fold increase in cell number; the 6A (*NOX1* knockdown) cells demonstrated lower levels of proliferation in the presence or absence of IL-4; the G6 cells (partial *NOX1* knockdown) displayed modest growth and modest enhancement of proliferation in the presence of IL-4. **E**. IL-4R expression is independent of NOX1 expression. Irrespective of NOX1 status, there was no significant change in IL-4Rα expression as measured by Western analysis (left panel) or quantitative RT-PCR (right panel). GAPDH and β-actin served as the internal controls, respectively. Data represent the mean ± SD of at least 3 experiments. ** = *P* < 0.01; *** = *P* < 0.001.

We recently reported that stable knockdown of NOX1 in HT-29 colon cancer cells (clone 6A with > 90% inhibition of NOX1; and clone Si6/G6 with ≈ 65% NOX1 knockdown) significantly decreases ROS formation compared to cells carrying a scrambled NOX1 shRNA (SC cells) or the parental line [16]. As demonstrated in Figure 2A, IL-4 exposure (50 ng/ml) for 24 h increased NOX1 protein expression in both parental HT-29 cells and scrambled shRNA control cells but had modest to no effect on NOX1 knockdown clonal lines, whether or not IL-4 exposure was performed in the presence or absence of 10% serum. Using a chemiluminescent probe to detect phorbol myristate acetate [PMA]-induced NOX1 activity, we found that treatment with IL-4 (50 ng/ml) for 24 h significantly increased ROS generation in both SC cells and the parental HT-29 line (*P* < 0.001); ROS formation in these two control cell lines was completely eliminated by treatment with superoxide dismutase [SOD] (Figure 2B). However, as was the case for NOX1 protein expression, NOX1 activity (ROS production) was not significantly increased following incubation with IL-4 for the 6A NOX1 knockdown cells; ROS production for the IL-4-treated Si6/G6 cells (that exhibit a lesser degree of NOX1 knockdown) was increased over control levels but much less so than scrambled controls or the parental line (Figure 2B). To confirm these results, we utilized a second measure of intracellular ROS production, the conversion of the non-fluorescent probe 2′,7′-dichlorodihydrofluorescein diacetate (H_2_-DCFDA) to the fluorescent moiety dichlorofluorescein (DCF) which can be measured by analytical cytometry [45]. We found that IL-4 treatment of parental HT-29 cells for 96 h produced a substantial right shift (increase) in green fluorescence consistent with increased ROS production; pre-incubation of HT-29 cells with the flavin dehydrogenase (and NOX) inhibitor DPI decreased ROS production (Figure 2C). In related experiments under identical conditions, other than for the use of a 48 h rather than a 96 h cytokine treatment time, exposure of HT-29 cells to IL-13 rather than IL-4 produced a very similar right shift in DCF fluorescence (data not shown). However, when HT-29 cells were treated with IL-4 (50 ng/ml) for 5 min (rather than 96 h), we could not demonstrate an increase in DCF fluorescence compared to colon cancer cells exposed to medium alone (Figure 2C).

Because stable NOX1 knockdown in HT-29 cells significantly decreases tumor cell proliferation [16], we examined the effect of IL-4 exposure on the growth of parental HT-29 cells and NOX1 knockdown clonal variants. Concordant with the effect of IL-4 on NOX1 expression and ROS production in these cell lines, IL-4 exposure significantly increased tumor cell proliferation in both parental HT-29 and scrambled shRNA control cells over a 96 h period of observation (*P* < 0.001), and modestly increased proliferation in Si6/G6 cells (Figure 2D). On the other hand, IL-4 produced no effect on the growth of 6A cells which possess minimal NOX1 protein (Figures 2D and 2E). Furthermore, treatment for 48 h with IL-4 did not affect the growth of CCD-112 normal colon cells that do not express NOX1 (data not shown). We also examined the level of IL-4Rα mRNA and protein in our panel of HT-29 cells to exclude the possibility that clonal selection might have altered receptor expression (and hence, sensitivity to IL-4); as demonstrated in Figure 2E, NOX1 knockdown had no effect on the expression of the IL-4Rα in our control HT-29 cells as well as HT-29-derived knockdown cells at either the mRNA or protein level.

To gain a better understanding of the kinetics of the IL-4-mediated modulation of NOX1, we also studied the effect of early elimination of IL-4 from the tissue culture medium on NOX1 expression and HT-29 cell proliferation (Supplementary Figure S2). When IL-4 was removed from the medium after 24 h, NOX1 expression continued to increase for another 24 h, but subsequently declined steadily with an approximate half-time of 48 h; however, 96 h following removal of IL-4, the NOX1 expression level was still above baseline. The decrease in NOX1 expression over time was mirrored by a decrease in the degree of cytokine-enhanced proliferation compared to continuous IL-4 exposure, albeit at a level that was still higher than in control cells exposed to medium alone. To evaluate the generality of these observations, we also examined the effect of IL-4 exposure on ROS production and tumor cell proliferation in DLD-1 human colon cancer cells (Supplementary Figures S1E and S1F). Increased NOX1 expression produced by IL-4 exposure in the DLD-1 line, similar to HT-29 cells, was also associated with an increase in tumor cell growth and the production of ROS.

Because IL-4 exposure has been shown previously to upregulate survivin and to alter the expression of several members of the apoptotic pathway [38, 39, 46], we evaluated whether IL-4-mediated changes in the apoptotic cascade might explain the enhanced proliferation rate we observed for HT-29 cells. We found, however, that exposure of HT-29 cells to IL-4 for either 24 or 96 h did not affect the expression of Bcl-2 or alter the integrity of caspase 3 or 8 (Supplementary Figure S2D).

### Functional ROS production by NOX1 is required to enhance colon cancer cell proliferation

**Figure 3:**
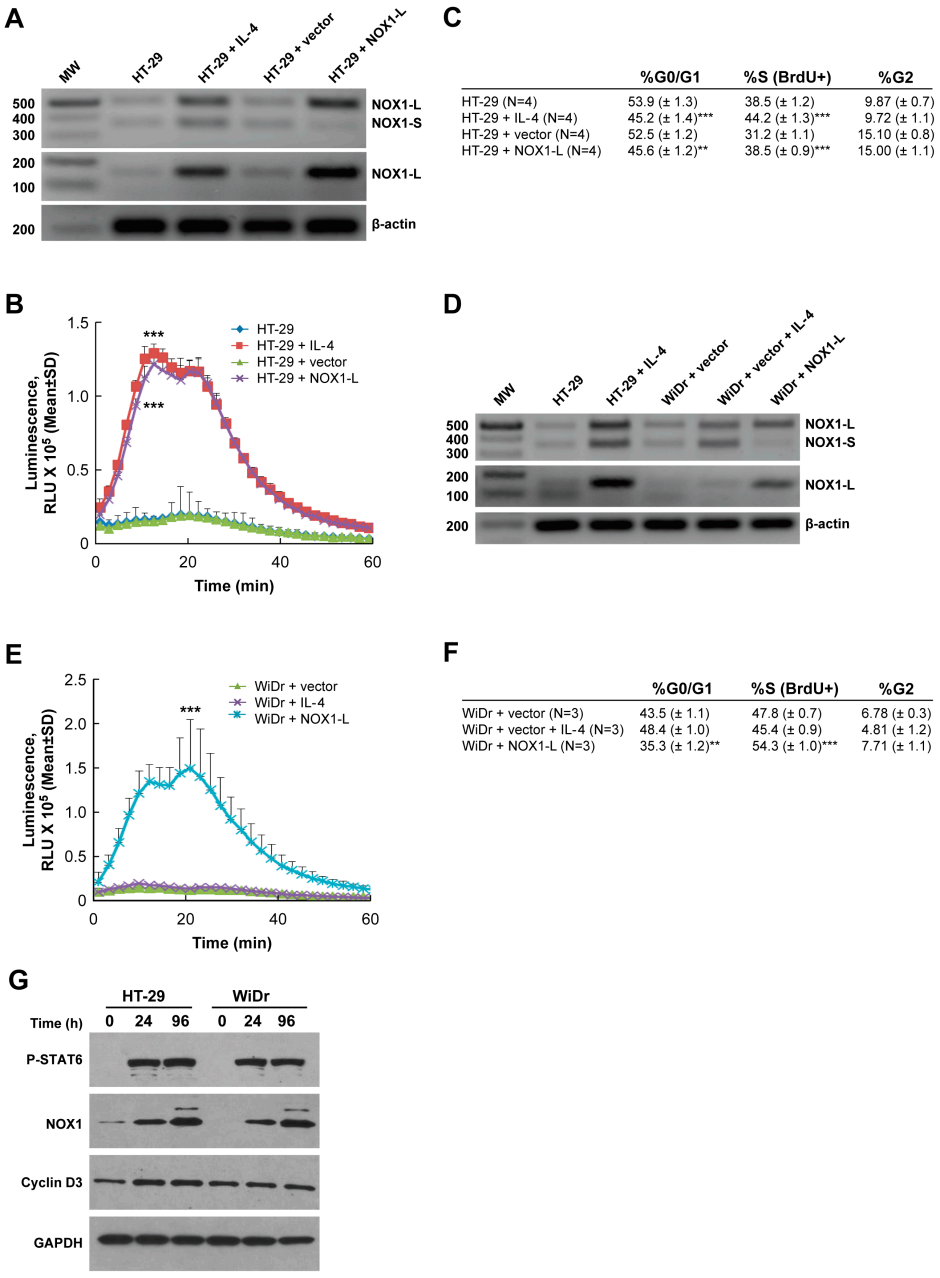
Functional activity of NOX1 correlates with colon cancer cell proliferation **A**. IL-4 exposure for 24 h increases expression of full-length *NOX1* in HT-29 cells. PCR and subsequent DNA gel analysis were performed to detect *NOX1-L* and *NOX1-S* in IL-4-treated HT-29 cells. A *NOX1-L* construct was transiently transfected into HT-29 cells and used as the control 48 h after transfection. **B**. IL-4 treatment (50 ng/ml for 24 h) supports PMA-stimulated superoxide production in HT-29 cells. HT-29 cells 48 h after transient transfection with a *NOX1-L* construct served as a positive control. **C**. Cell cycle analysis was performed using analytical cytometry following BrdU labeling of HT-29 cells that had been treated for 24 h with 50 ng/ml of IL-4. HT-29 cells evaluated 48 h after transient transfection with *NOX1-L* were used as the control. The percentage of cells in the different phases of the cell cycle is shown in the table. **D**. IL-4 exposure (50 ng/ml for 24 h) increases the expression of *NOX1* in WiDr cells. PCR and subsequent DNA gel analysis were performed to detect *NOX1-L* and *NOX1-S* in WiDr cells treated with IL-4. Transient transfection of *NOX1-L* into WiDr cells served as the control. **E**. *NOX1-L* rather than *NOX1-S* supports PMA-stimulated superoxide production in WiDr cells. WiDr cells treated with IL-4 (50 ng/ml for 24 h) or 48 h after transient transfection with a *NOX1-L* construct were evaluated for the extent of PMA-induced superoxide production. **F**. Cell cycle analysis was performed using analytical cytometry following BrdU labeling of WiDr cells that had been treated for 24 h with 50 ng/ml of IL-4. WiDr cells evaluated 48 h after transient transfection with *NOX1-L* were used as the control. The percentage of cells in the different phases of the cell cycle is shown in the table. **G**. Western analysis was performed on HT-29 and WiDr cells treated with IL-4 (50 ng/ml) for variable periods of time (0, 24, 96 h). Activated STAT6 and NOX1 levels were examined as indicators of IL-4 efficacy in these experiments; GAPDH served as the loading control. All data represent the mean ± SD of at least three experiments. *** = *P* < 0.001.

To explore potential mechanism(s) of IL-4-related tumor cell proliferation further, we examined the effect of IL-4 on the expression of the two known isoforms of *NOX1, NOX1-L* and *NOX1-S* [47]. Two sets of primers were used to detect these two distinct isoforms; *NOX1-L* is the full-length, functional form of NOX1, while *NOX1-S* is a smaller splice variant that lacks an NADPH binding site. RT-PCR analysis demonstrated that IL-4 treatment for 24 h increased the expression of *NOX1-L* in HT-29 cells which was associated with a marked increase in PMA-induced ROS formation (Figure 3A and 3B). Transient transfection of HT-29 cells with *NOX1-L* (Figure 3A) increased ROS production by HT-29 cells 48 h following transfection to a degree equivalent to that observed following IL-4 treatment (Figure 3B). When the effect of IL-4 exposure on cell cycle progression was examined in HT-29 cells using BrdU labeling and analytical cytometry, treatment with IL-4 for 24 h significantly increased the fraction of HT-29 cells in S phase (by > 5%; *P* < 0.001; Figure 3C). Consistent with our measurements of reactive oxygen production, transient overexpression of *NOX1-L* also increased cell cycle traverse through S phase by > 5% 48 h following transfection (Figure 3C; *P* < 0.001). In concert with these results, exposure of DLD-1 cells to IL-4 primarily increased *NOX1-L* expression (Supplementary Figure S1G), which was associated with increased tumor cell proliferation and ROS production (Supplementary Figures S1E and S1F).

However, when WiDr cells were treated with IL-4 for 24 h, *NOX1-L* did not appear to be specifically induced, which led to no discernable effect of IL-4 on reactive oxygen production or cell cycle progression (Figures 3D, 3E, and 3F). On the other hand, if WiDr cells were transiently transfected with *NOX1-L*, production of ROS 48 h after transfection was significantly increased (*P* < 0.001), as was the S-phase fraction (Figures 3E and 3F). Finally, we found that in HT-29 cells (but not WiDr cells), upregulation of functional NOX1 was associated with a modest increase in the expression of cyclin D_3_, which could help to explain the enhanced cell cycle traverse through G_1_ that we observed (Figure 3G). Taken together, these results suggest that enhanced expression of *NOX1-L* promotes ROS generation that is associated with an increase in cell cycle progression in human colon cancer cells.

### Induction of NOX1 expression by IL-4 or IL-13 requires the presence of functional IL-4Rα

**Figure 4:**
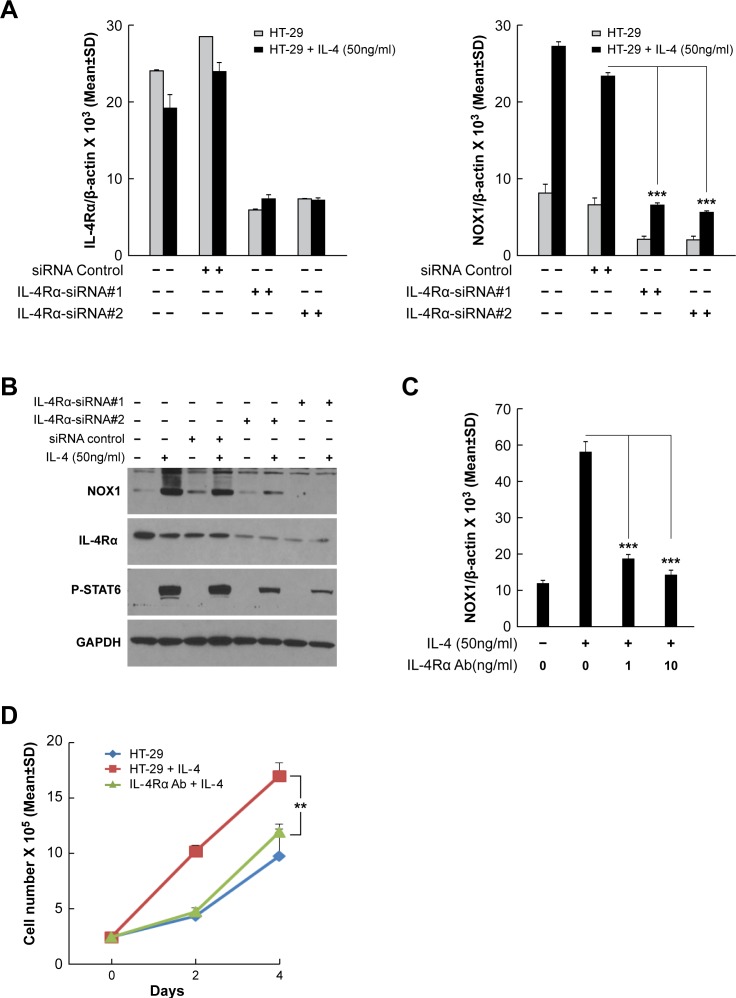
Inhibition of IL-4R blocks IL-4-induced NOX1 expression **A**.-**B**. Knockdown of IL-4Rα blocks NOX1 expression at the mRNA **A**. and protein **B**. levels. HT-29 cells were transiently transfected with either of the two different IL-4Rα specific siRNAs and treated with IL-4 (50 ng/ml) 24 h later. Cells were harvested 24 h post-treatment and examined by quantitative RT-PCR **A**. or by Western analysis **B**.. β-actin and GAPDH served as the internal controls for RT-PCR or Western analysis, respectively. **C**. IL-4-induced NOX1 expression requires the presence of IL-4R. IL-4-induced NOX1 expression was abolished in HT-29 by the concurrent presence of an IL-4Rα neutralizing antibody in the absence of FBS, as shown by quantitative RT-PCR. **D**. IL-4 stimulates cell proliferation through the involvement of IL-4R. IL-4Rα antibody (1 ng/ml) significantly inhibited IL-4-enhanced HT-29 proliferation. Data represent the mean ± SD of three experiments. ** = *P* < 0.01; *** = *P* < 0.001.

To examine the requirement for functional IL-4 or IL-13 binding to the Type II IL-4R in the induction of NOX1 expression, IL-4Rα expression was knocked down with two different small interfering RNAs [siRNAs] (Figure 4A and Supplementary Figure S3A). We found that inhibition of IL-4Rα expression significantly decreased IL-4- or IL-13-stimulated NOX1 expression in HT-29 cells at both the mRNA (*P* < 0.001) and protein levels (Figures 4A and 4B; Supplementary Figure S3A). When HT-29 cells were pretreated with a neutralizing mouse anti-human monoclonal antibody against the IL-4Rα chain (R & D Systems; Clone 25463), prior to treatment with IL-4 or IL-13, cytokine-enhanced NOX1 expression was significantly decreased (Figures 4C and Supplementary Figure S3B; *P* < 0.001). As shown in Figure 4D, the simultaneous presence of the neutralizing antibody and IL-4 blocked IL-4-enhanced proliferation of HT-29 cells (*P* < 0.01). These results indicate that the Type II IL-4R is required for IL-4 or IL-13-mediated NOX1 upregulation and also suggest that IL-4 and IL-13 could share the same signaling pathway to induce NOX1 expression.

### JAK1/STAT6 signaling pathway is involved in the induction of NOX1 by IL-4 and IL-13

**Figure 5:**
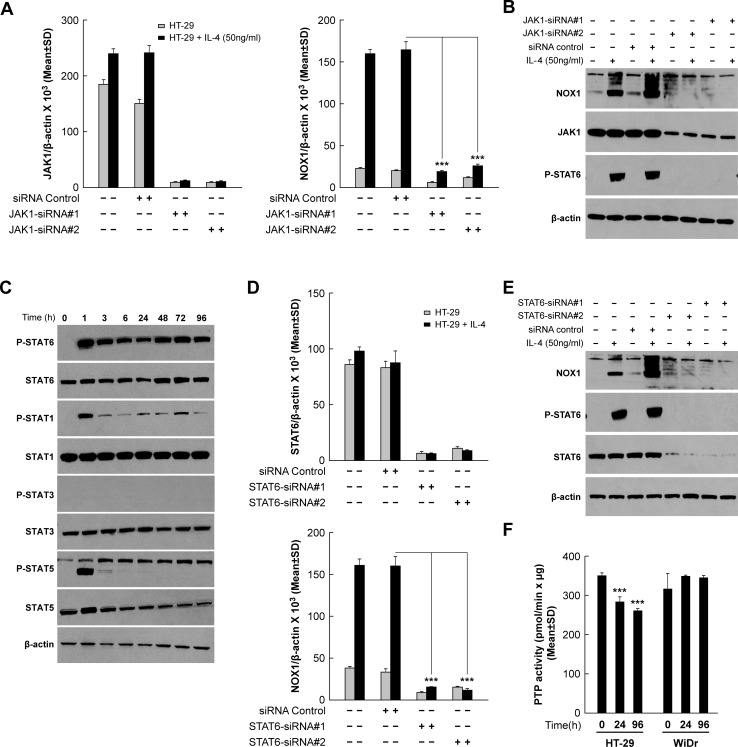
JAK1/STAT6 activation is required for IL-4-induced NOX1 expression **A**.-**B**. JAK1 knockdown blocks IL-4-mediated enhancement of NOX1 expression at the mRNA **A**. and protein levels **B**.. HT-29 cells were transiently transfected with either of the two different JAK1-specific siRNAs and treated with IL-4 (50 ng/ml) 24 h later. Cells were harvested at 24 h post-treatment and examined by quantitative RT-PCR **A**. and Western analysis **B**.. β-actin served as the internal control. **C**. IL-4 treatment results in a transient increase in phosphorylated STAT1 and STAT5, and a sustained increase in phosphorylated STAT6. Western analysis was performed on IL-4 (50 ng/ml)-treated HT-29 cells harvested at different time points following initiation of IL-4 exposure. β-actin served as the loading control. (**D**.-**E**.) STAT6 knockdown blocks IL-4-related stimulation of NOX1 expression at the mRNA **D**. and protein levels **E**.. HT-29 cells were transiently transfected with either of two different STAT6-specific siRNAs and treated with IL-4 24 h later. Cells were harvested at 24 h post-treatment and examined by quantitative RT-PCR **D**. and Western analysis **E**.. **F**. PTP activity was evaluated in HT-29 and WiDr cells following exposure to IL-4 (50 ng/ml) for the indicated times. PTP levels were determined by measuring the dephosphorylation of a standard phosphopeptide using malachite green as the detector. β-actin served as the internal control. Data represent the mean ± SD of three experiments. *** = *P* < 0.001.

Three members of the Janus Kinase family, JAK1, JAK2, and JAK3, have been reported to associate with components of the IL-4R complex [27]. We found that JAK1 was the major Janus Kinase family member expressed in HT-29 cells (Supplementary Figure S4A). When JAK1 was silenced with two independent siRNAs, IL-4-induced NOX1 expression was decreased at both mRNA (*P <* 0.001; Figure 5A) and protein levels (Figure 5B). Knockdown of JAK2 had no effect on NOX1 expression that was increased by IL-4 (Supplementary Figure S4B). Unfortunately, lack of a phospho-specific JAK-1 antibody impeded determination of the critical tyrosine residues required for JAK-1 activation. We next examined whether members of the STAT family were activated by IL-4, since STAT6 has been reported previously to play an important role in signal transduction associated with IL-4 exposure [40]. Treatment of HT-29 cells with IL-4 was followed by a rapid increase in the phosphorylation of STAT1, 5, and 6 (but not STAT3) (Figure 5C); however, only phosphorylated STAT6 (p-STAT6), which is phosphorylated by the receptor associated kinase JAK1, remained highly activated for the full 96 h of cytokine exposure. STAT6 phosphorylation was blocked by JAK1 specific siRNAs (Figure 5B), suggesting that STAT6 is downstream of JAK1 and may be involved in the IL-4-related increase in NOX1 expression. STAT6 knockdown experiments confirmed the involvement of this transcription factor in IL-4-induced NOX1 expression (Figures 5D and 5E). In related experiments, we found that both JAK1 and STAT6 played an important role in modulating IL-13-related NOX1 expression (Supplementary Figures S4C and S4D). These results strongly suggest that JAK1 and STAT6 can mediate enhanced expression of NOX1 produced by IL-4 and IL-13, consistent with previous data suggesting that STAT6 is a principal STAT activated in response to IL-4 stimulation, where it plays a critical role in modifying the expression of IL-4-responsive genes [48]. We hypothesize that the prolonged activation of STAT6, furthermore, might have been enhanced by ROS-related inhibition of redox sensitive PTP moieties (observed for the HT-29 line but not for WiDr cells that lack functional NOX1) (Figure 5F). It is important to point out in this regard that beyond activating the JAK/STAT pathway, IL-4 and IL-13 can increase MAPK (ERK1/2) signaling in a fashion that depends on NOX1-related ROS formation and is associated with altered phosphatase function [26].

### IL-4-enhanced NOX1 expression depends on the activation of GATA3 by S308 phosphorylation

**Figure 6:**
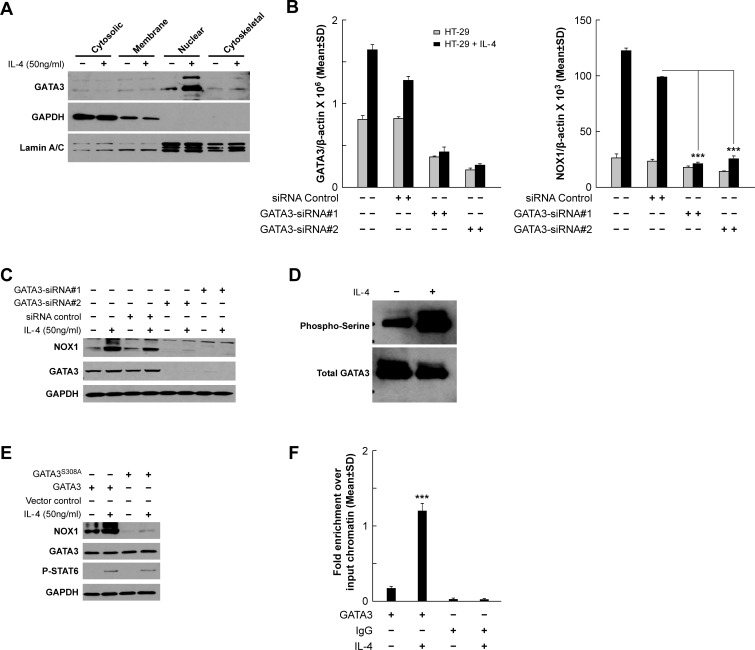
GATA3 plays a role in the transcriptional regulation of NOX1 **A**. IL-4 triggers GATA3 nuclear translocation demonstrated by Western analysis of HT-29 cells treated for 24 h with IL-4. GAPDH and Lamin A/C were used as the cytosolic and the nuclear protein loading controls, respectively. **B**.-**C**. GATA3 knockdown blocks increased NOX1 mRNA expression following IL-4 stimulation (50 ng/ml) at the mRNA **B**. and protein levels **C**.. HT-29 cells were transiently transfected with either of two different GATA3-specific siRNAs and treated with IL-4 24 h later. Cells were harvested 24 h following IL-4 treatment and examined by quantitative RT-PCR **B**. and Western analysis **C**.. β-actin and GAPDH served as the internal controls. **D**. The serine residues of GATA3 are phosphorylated following IL-4 stimulation. Immunoprecipitation of GATA3 and subsequent Western analysis to determine the extent of serine phosphorylation were performed on HT-29 cells treated with solvent or IL-4 (50 ng/ml for 24 h). **E**. Serine-phosphorylation of GATA3 is a prerequisite for IL-4-induced NOX1 expression. Western analysis was performed on HT-29 cells expressing either intact GATA3 or GATA3^S308A^. **F**. Fragments corresponding to the *NOX1* promoter region, pulled down with a GATA3 antibody, are enriched following treatment with 50 ng/ml of IL-4 for 24 h. Chromatin immunoprecipitation assay and subsequent quantitative PCR were performed on HT-29 cells exposed to IL-4 (50 ng/ml) or solvent. Isotype-matched IgG was used as the control. Data represent the mean ± SD of three experiments. *** = *P* < 0.001.

Although IL-4 enhanced the phosphorylation of STAT6, and STAT6 knockdown blocked the induction of NOX1 expression in HT-29 cells, we could not define consensus STAT6 binding sequences in the NOX1 promoter. This observation suggested that other transcription factors might be involved that could link STAT6 activation with the induction of NOX1 expression. Previous studies of CD4^+^ T cell differentiation indicated that GATA3 was selectively induced by IL-4 following STAT6 activation [49]. Upon further investigation, we found that the NOX1 promoter contained four possible GATA3 binding sites. As demonstrated in Figure 6A, IL-4 treatment increased the nuclear expression of GATA3; the time course of STAT6 phosphorylation and increased GATA3 expression in the nucleus suggested that activation of STAT6 preceded that of GATA3 (Supplementary Figure S5A). Silencing GATA3 with two independent siRNAs blocked IL-4- or IL-13-enhanced NOX1 mRNA expression (Figure 6B, *P <* 0.001; Supplementary Figure S6A, P < 0.001) as well as baseline and IL-4-enhanced protein expression (Figure 6C). These results suggest that GATA3 is an activator of NOX1 expression following IL-4 or IL-13 stimulation.

Because phosphorylation of GATA3 appears to be required for its translocation to the nucleus and subsequent effects on gene transcription [50, 51], we examined whether GATA3 phosphorylation was necessary for IL-4-induced NOX1 expression. Immunoprecipitation experiments revealed that IL-4 treatment increased the phosphorylation of serine residues on GATA3 in HT-29 cells compared to vehicle-treated controls (Figure 6D). Transient overexpression of GATA3 increased IL-4-induced NOX1 expression in HT-29 cells (Figure 6E). However, HT-29 cells transiently transfected with a GATA3 construct carrying a mutation at S308A failed to induce NOX1 expression following IL-4 treatment (Figure 6E). These results support a role for serine phosphorylation of GATA3 in the mechanism of IL-4-induced NOX1 expression.

### Transcriptional regulation by GATA3 plays a major role in the induction of NOX1 expression by IL-4 and IL-13

**Figure 7:**
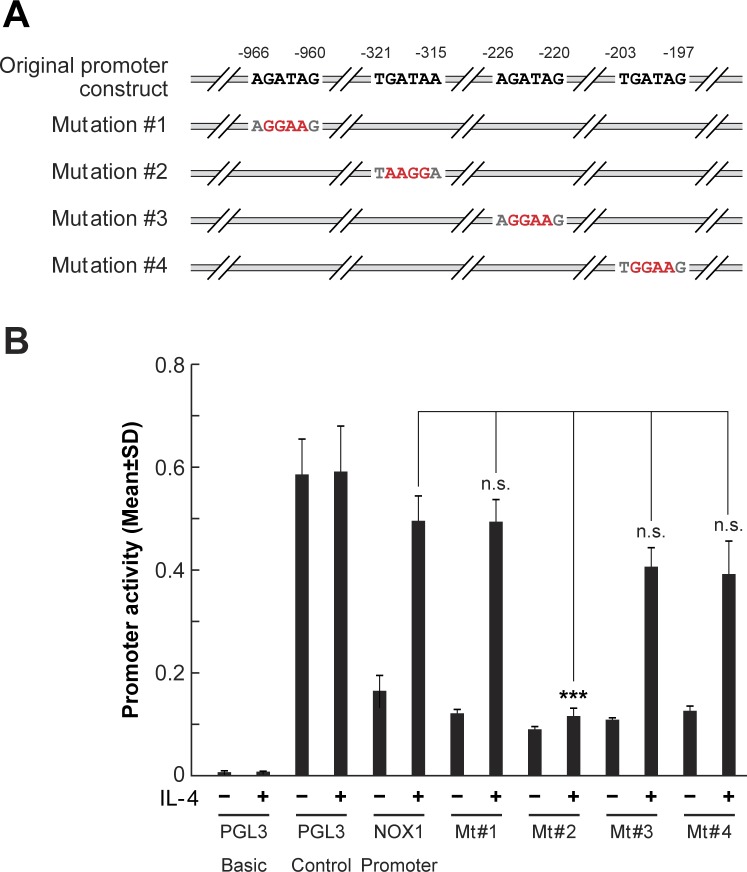
GATA3 binds to NOX1 promoter region -321/−315 **A**. Schematic illustration of the *NOX1* promoter region and its variants generated by mutagenesis, highlighting the potential GATA3 binding sites. Mutated binding sites are shown in red. These promoter regions were individually constructed as PGL3 reporters. **B**. Mutation at the -321/−315 region abolishes luciferase activity associated with IL-4-mediated NOX1 promoter activity. HT-29 cells were transfected with the individual reporter constructs in the presence or absence of IL-4 (50 ng/ml), and luciferase activity was measured 48 h later. Data represent the mean ± SD of three experiments. *** = *P* < 0.001; n.s. = not significant.

Using promoter-reporter assays, we sought to define whether GATA3 binding to the NOX1 promoter played a critical role in IL-4-dependent upregulation of NOX1 expression. In the presence of IL-4, reporter gene transcription, from a 960-bp segment corresponding to -179 to -1139 bps of the promoter sequence upstream from the NOX1 transcription start site (Figure 7A), increased luciferase activity ≈ 4-fold in HT-29 cells (Figure 7B). Further analysis of this sequence predicted four putative GATA binding sites: -966/−960, -321/−315, -226/−220, and -203/−197. To test the potency of these predicted binding sites to induce NOX1 transcription following IL-4 exposure, we developed individual mutants of each potential GATA binding site (Figure 7A). When the GATA3 cis-element, -321/−315, was mutated (mutation #2), NOX1 promoter-reporter gene transcription was completely abolished (Figure 7B), supporting the involvement of GATA3 in IL-4-enhanced NOX1 expression, as well as the involvement of the -321/−315 GATA3 cis-element. In contrast, mutations in the other three GATA family cis- elements did not influence NOX1 promoter-reporter gene transcription in the presence of IL-4. These results suggest that the conserved consensus sequence required for the binding of GATA3 is -321/−315. We also performed chromatin immunoprecipitation (ChIP) assays to detect GATA3 binding to the NOX1 promoter. The primers utilized were specific for the possible GATA3 binding site defined by the mutation studies: bp -321/−315. Our results demonstrated that GATA3 binding was significantly increased following treatment with either IL-4 or IL-13 (Figure 6F; Supplementary Figure S6B). Furthermore, STAT6 knockdown inhibited the binding of GATA3 to the NOX1 promoter site, suggesting that GATA3 responds to STAT6 activation upon IL-4 exposure (Supplementary Figure S5B). Taken together, these data provide evidence demonstrating that IL-4 and IL-13 upregulate NOX1 in colon cancer cells though the IL-4R/STAT6 pathway, and that GATA3 plays an important role in the transcriptional regulation of NOX1.

### NOX1 expression in human colon cancers and its relationship to IL-4R expression in colon cancer and adjacent colonic epithelium

**Figure 8:**
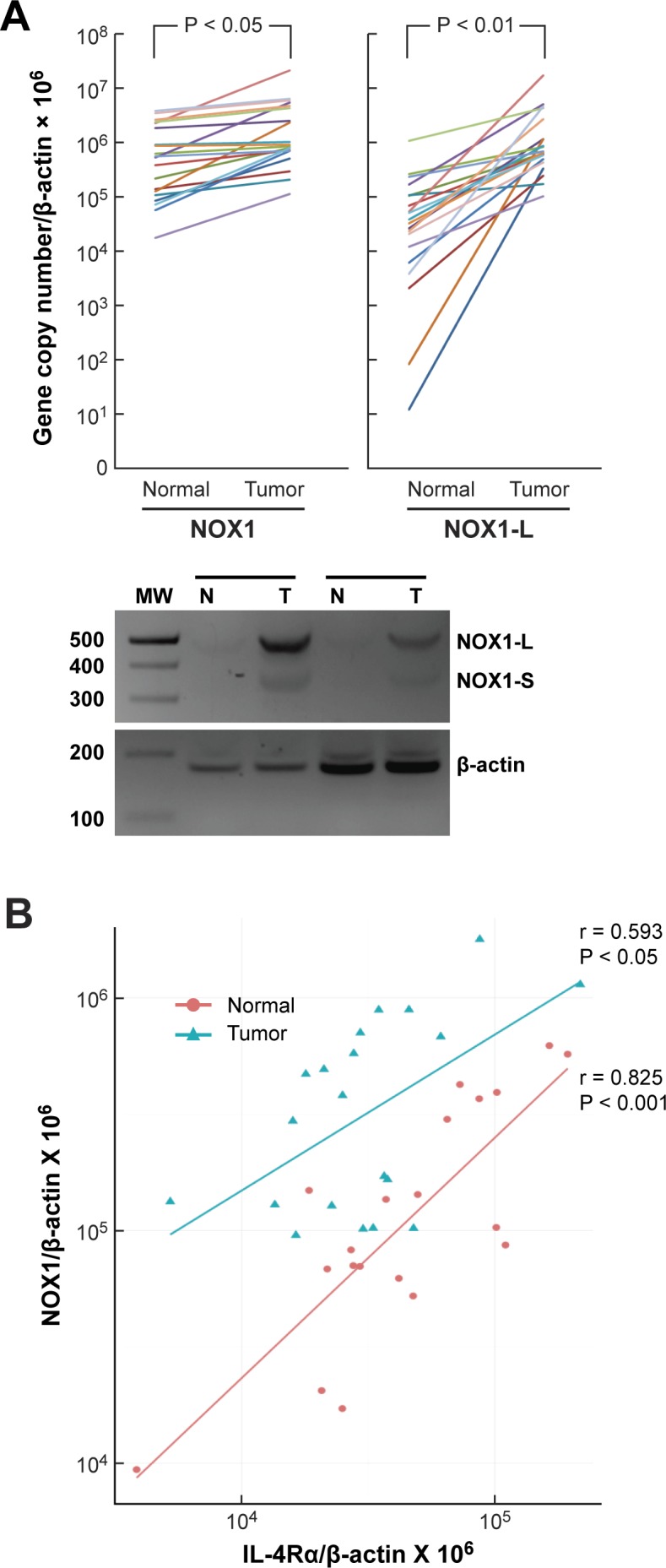
NOX1 expression in human colon cancers and adjacent uninvolved colonic epithelium and its relationship to IL-4Rα expression **A**. Twenty pairs of human colon cancer surgical specimens and adjacent normal colonic epithelium were analyzed by quantitative RT-PCR for the expression of NOX1 and its functional isoform NOX1-L. β-actin served as the control. Paired, two-tailed *t* tests were used to evaluate the results; the Wilcoxon signed rank test was also used to test for differences in expression level between tumor and normal samples. The likelihood of our observations occurring if there was truly no difference in the expression between tumor and normal samples is < 0.0001. The lower panel shows the results of two representative patient samples in which cDNA was cloned from the surgical samples and subjected to PCR to examine the abundance of different NOX1 isoforms (NOX1-L and NOX1-S). **B**. NOX1 and IL-4α expression levels were examined by quantitative RT-PCR using the tumors (blue) and the respective adjacent normal tissues (red) of 20 colon cancer patients. β-actin served as the internal control. Pearson's correlation coefficient (r) and level of significance (*P*) using a two-tailed *t* test for both groups are shown in the figure.

To examine the clinical relevance of our finding that in colon cancer cell lines IL-4 upregulates the expression of a functional *NOX1-L* isoform, we measured NOX1 expression in twenty tissue pairs consisting of surgically-resected colon cancers and their adjacent normal colonic mucosae. We found that mRNA levels of total NOX1, as well as the NOX1-L isoform, were significantly higher in tumors compared to uninvolved, adjacent colonic epithelium (Figure 8A). Furthermore, there was a significant relationship in both tumor samples and normal tissues between levels of IL-4Rα and NOX1 mRNA (Figure 8B). The relationship between IL-4Rα and NOX1 in these tumors and adjacent normal tissues suggests that the conditions exist in the clinic to support a mechanistic relationship between IL-4 and colon cancer proliferation.

## DISCUSSION

A growing body of evidence supports a role for the T_H_2-cytokines IL-4 and IL-13 in the pathogenesis of pre-malignant chronic inflammatory diseases of the gastrointestinal tract [52–55] and in the enhancement of colorectal cancer cell proliferation [31, 39, 56]. Furthermore, recent studies have demonstrated that IL-4 and IL-13, as well as the Type II receptor they share, are expressed widely in human colorectal cancers [31, 57], as well as in a variety of other epithelial malignancies [35, 37]. Enhanced cellular proliferation in the presence of IL-4 or IL-13 has, for the most part, been attributed to increased JAK/STAT signaling (primarily through STAT6) [48] or to the upregulation of the expression of survivin and other anti-apoptotic proteins, including Bcl-2 and Bcl-xL [39, 46].

For the experiments reported herein, we explored another potential mechanism of IL-4- or IL-13-mediated growth control: the generation of ROS. We found that both cytokines increased the expression of NOX1 (but not other NOX species) over a period of 12 to 48 h in a panel of human colorectal cancer cell lines that possess the Type II IL-4R. The extent of functional NOX1 expression in HT-29 and DLD-1 human colon cancer cells, as measured by the generation of ROS (Figures 2B and 2C; Supplementary Figure S1B and S1F) correlated well with the degree of tumor cell growth stimulation by IL-4 or IL-13; in the presence of near-complete NOX1 knockdown (which had no effect on the expression of the Type II IL-4R), IL-4 exposure did not affect the growth rate of the tumor cells (Figure 2D). Enhanced cellular proliferation was dependent upon the presence of a functional Type II IL-4R (Figure 4D) and involved an acceleration of cell cycle progression through S-phase (Figure 3C).

These results mirror our recent demonstration of the effects of stable NOX1 knockdown with shRNA in HT-29 cells [16]; in those experiments, we found that inhibition of NOX1 expression (and consequently of ROS formation) in the HT-29 line produced a profound block in cell cycle progression at the G_1_ interface (related to diminished cyclin D_1_ expression), leading to a significant decrease in tumor cell proliferation. We have also shown that inhibition of NOX1 activity with diphenylene Iodonium [DPI] or 2-di-thienyl Iodonium [DTI] also induced a G_1_ block in HT-29 cells with an associated decrease in tumor cell growth [17]. In related studies by other laboratories, marked G_1_ delay has been demonstrated following exposure of fibroblasts to the ROS scavenger N-acetyl-L-cysteine [58] or following overexpression of catalase (which detoxifies H_2_O_2_) in murine aortic endothelial cells [59]. Thus, demonstration of enhanced BrdU incorporation in HT-29 and WiDr colon cancer cells following IL-4 exposure or transient transfection of NOX1-L, respectively, is consistent with ROS-related regulation of cyclin D_3_ levels in our colon cancer lines, and is in harmony with a previous study of the effect of NOX1 levels on cyclin D_1_ expression in murine lung epithelial cells [60].

However, our results differ, at least in part, from a previous report that examined the effect of IL-4 [41] on reactive oxygen production by human tumor cells. In this study, cytokine exposure was reported to induce immediate (1-15 min), ligand-dependent ROS production that required the presence of NOX1 (and potentially other NADPH oxidases including NOX4, NOX5, and DUOX2). Because of these differences, we performed control experiments to examine the time course of ROS production following IL-4 exposure in human colorectal cancer cells. As shown in Figure 2C, we found no evidence of an immediate burst of ROS production, as measured by DCF fluorescence, following cytokine exposure in HT-29 cells. These results are consistent with a previous study from our laboratory which demonstrated by real time RT-PCR that the constitutive expression levels of all seven NOX homologues in the A549 human lung cancer line were at or below the lower limit of detection for our PCR assay [61].

It should also be pointed out that some previous studies have reported that IL-4 is growth inhibitory for certain colorectal cancer cells, including the HT-29 cell line [62, 63], while stimulating tumor cell proliferation in other tumor cell types [63]. Specific activation of STAT1 with the induction of p21-dependent growth arrest was proposed as the mechanism of growth inhibition in the study by Chang and colleagues [63]. However, as demonstrated in Figure 5C, we found that exposing HT-29 cells to IL-4 produced a profound and prolonged (96 h) activation of STAT6, but only transient (1-3 h) phosphorylation of STAT1, that was associated with IL-4-dependent, enhanced cell cycle progression. Furthermore, when IL-4 was administered to patients with a variety of solid tumors in prior clinical trials, no meaningful therapeutic activity was demonstrated [64]. These results, in concert with evidence that IL-4 stimulates the growth of primary colon cancer cell and normal enterocyte growth (in addition to human colorectal cancer cell lines), suggest that the preponderance of evidence favors an important role for the proliferative potential of IL-4 [38] in human cancers [39].

In addition to increasing NOX1-dependent cell cycle progression, as reported in this study, IL-4 has been demonstrated to stimulate tumor cell proliferation by enhancing the expression of anti-apoptotic proteins and/or signaling through the MAPK pathway [40]. We found in previous experiments [26] that IL-4-related MAPK signaling may be mediated by NOX1-dependent ROS production. Based on those results, it could be hypothesized that the enhanced proliferation we observed in parental and scrambled shRNA-containing HT-29 cells following IL-4 exposure (but not in NOX1 knockdown cells) may be a downstream consequence of ROS-related ERK activation on the cell cycle regulatory apparatus [65]. Although upregulation of anti-apoptotic proteins undoubtedly contributes to IL-4-induced growth enhancement in several model systems [39], such an effect of IL-4 does not appear to fully explain our results with HT-29 cells (Supplementary Figure S2D).

The regulation of NOX1 activity has traditionally been attributed to variations in the association or phosphorylation of members of the NOX1 complex (NOXO1, NOXA1, p22^phox^, and Rac) [11]. However, studies of NOX1 expression in Caco-2 human colorectal cancer cells demonstrated that binding of the transcription factor GATA6 to the NOX1 promoter played an important role in the regulation of NOX1 expression [66, 67]. In contrast, initiation of *IL-4* transcription in naïve T cells is regulated by GATA3 [29]; and the expression of GATA3 is significantly increased in the bowel mucosae of children with the inflammatory bowel disease, ulcerative colitis [68]. Thus, we evaluated potential GATA3 binding sites in the HT-29 cell promoter, and found by mutational analysis that the conserved consensus sequence required for the binding of GATA3 to the NOX1 promoter is located at bp -321/−315. These results support the possibility that GATA3 could be important for the regulation of NOX1 expression by pro-inflammatory cytokines [69].

To investigate the clinical relevance of our findings, we examined the relationship between expression levels of NOX1 and IL-4Rα in human colorectal cancer surgical specimens and adjacent uninvolved bowel mucosa by real time RT-PCR. At the mRNA level, there was a significant correlation between the two (Figure 8B). This observation was confirmed using a publicly available dataset that examined gene expression in colon cancers and associated normal tissues by expression array profiling [70] (data not shown). These data are consistent with the hypothesis that IL-4 or IL-13, generated in either an autocrine or paracrine fashion or by inflammatory cells, could be involved in activating STAT6-dependent upregulation of NOX1 expression in both colon cancers and adjacent bowel. Our prior results demonstrated that NOXO1 and NOXA1, in addition to NOX1, are also increased in clinical colon cancer specimens [61], facilitating the potential for active NOX1 activity. Because previous studies have documented the ability of NOX1-dependent ROS to produce genetic instability in mammalian cells [71], it is reasonable to suggest that the IL-4- or IL-13-mediated upregulation of NOX1 could play a role in the development of the colorectal cancers that occur in patients with inflammatory bowel disease or stimulate the progression of established colonic neoplasms.

**Figure 9:**
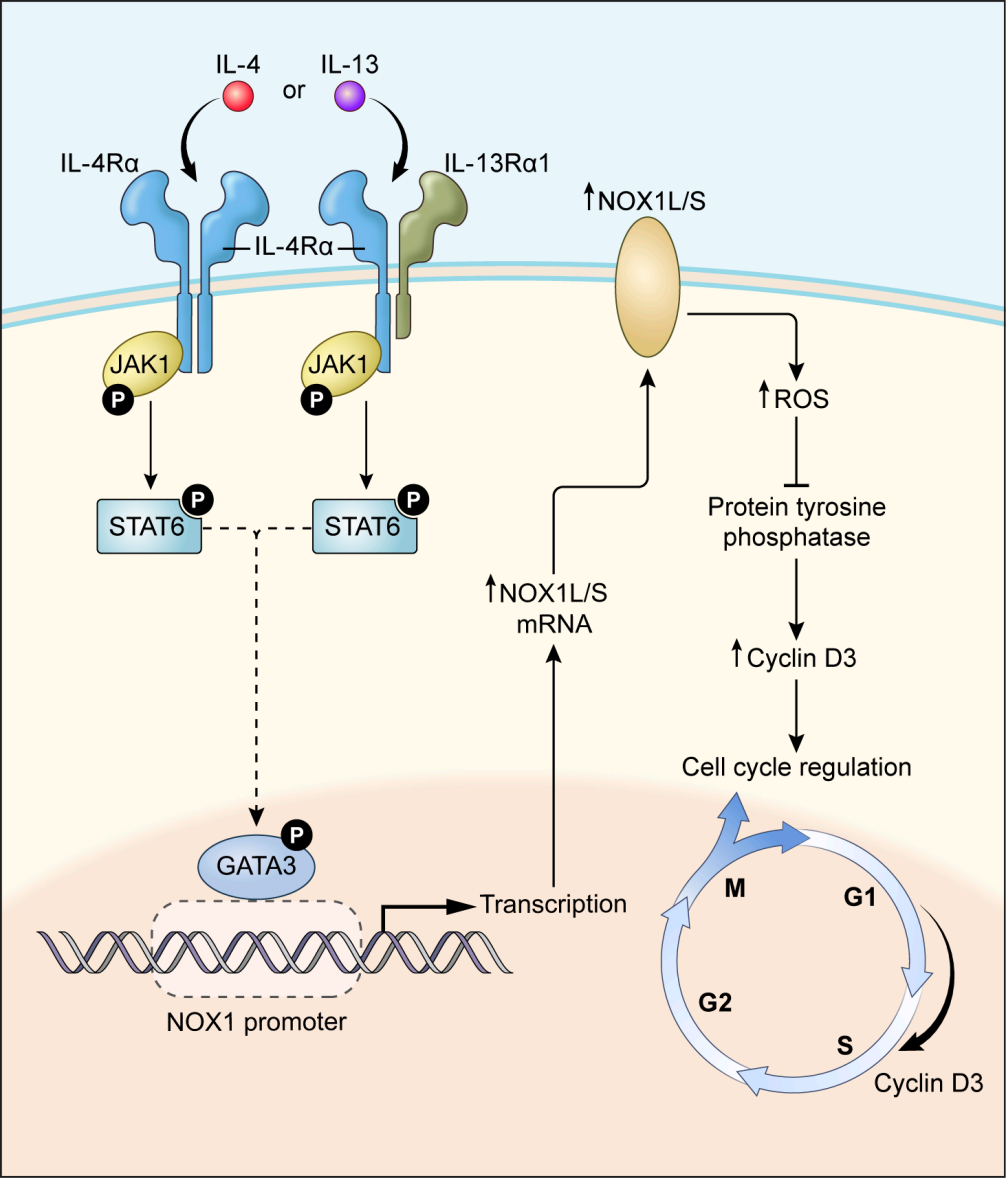
A proposed model for IL-4/IL-13-induced NOX1 expression and colon cell proliferation IL-4/IL-13 bind to the Type II IL-4 receptor activating the JAK1/STAT6 signaling pathway, triggering GATA3 nuclear translocation that drives NOX1 expression. GATA3 binding to the NOX1 promoter initiates transcription that is dependent on serine phosphorylation (S308). Increased expression of full-length NOX1 (NOX1-L) promotes ROS production which can inhibit PTP activity and increase cyclin D3 levels, leading to enhanced cell cycle traverse through S-phase and increased colon cancer cell proliferation. Human colon cancers, when compared to adjacent uninvolved colonic epithelia, demonstrate significantly higher levels of NOX1 and IL-4Rα mRNA, consistent with this model.

In summary, our studies (summarized in Figure 9) demonstrate that the T_H_2 cytokines IL-4 and IL-13 significantly enhance the transcription of NOX1, leading to the production of substantial amounts of O_2_^·¯^ by human colon cancer cells, and that increased NOX1 expression depends on the activation and binding of GATA3 to the NOX1 promoter. Furthermore, NOX1-dependent ROS are actively involved in stimulating tumor cell proliferation that is related, in part, to an increase in cell cycle progression through S-phase. In human colon cancers and adjacent normal tissues, the expression of NOX1 and the Type II IL-4R are significantly correlated. In light of these data, support exists for the possibility that NOX1-related ROS, produced as a consequence of IL-4 or IL-13 exposure, may play an important role in the development of a pro-oxidant milieu in the colon, conducive to colorectal carcinogenesis and/or malignant progression. In this context, the development of therapeutic approaches to interdict the pro-oxidant effects of IL-4 or IL-13, through a neutralizing antibody [72], other protein scaffolds [53], or direct inhibition of NOX1 [17] might provide a novel method to interfere with pro-inflammatory oxidative damage in the colon.

## MATERIALS AND METHODS

### Materials

Recombinant human IL-4 (catalog no. 204-IL-050) and recombinant human IL-13 (catalog no. 213IL-025) were purchased from R & D systems (Minneapolis, MN). *Antibodies*: Antibody against human β-actin (catalog no. A3853) was acquired from Sigma-Aldrich. Antibodies against human GAPDH and Lamin A/C were obtained from Cell Signaling Technology (catalog nos. 5174 and 4777). An antibody raised against human IL-4Rα (catalog no. MAB230) was from R & D Systems. Human GATA3 antibody was purchased from Santa Cruz Biotechnology (catalog no. sc-269). Human anti-phosphoserine antibody (catalog no. ab6639) and a second human IL4Rα antibody (catalog no. ab50277) were from Abcam Biochemical. The IL-4Rα antibody from Abcam was used for Western analyses; the R&D antibody was used for studies of IL-4Rα inhibition. Human anti-STAT6 antibody (catalog no. 611290) and human anti-phospho STAT6 (pY641; catalog no. 611566) were obtained from BD Biosciences. Antibodies against Bcl-2, caspase 8, pro-caspase 3, and cleaved caspase 3, and cyclins D_1_ and D_3_ were obtained from Cell Signaling Technology (catalog nos. 2872, 9746, 9662, 9661, respectively, and the cell cycle regulation antibody sampler kit catalog no. 9932). Anti-ATPase (catalog no. CS3010) and human anti-JAK1 (catalog no. CS3332) antibodies were purchased from Cell Signaling. A mouse monoclonal antibody against NOX1 was raised against a recombinant protein that represented 341 carboxy-terminal amino acids (224-564 amino acid sequence) of the human NOX1 protein, as previously described [73]. *Human primers*: NOX1 (catalog no. Hs00246589_m1), β-actin (catalog no. Hs99999903_m1), NOXA1 (catalog no. Hs00611456_g1), NOXO1 (catalog no. Hs00376039_g1), NOX2-5, DUOX1 and DUOX2 (catalog nos. Hs00166163_m1; Hs00571343_CE; Hs00110468_CE; Hs00674976_CE; Hs00719583_CE; Hs00647818_CE), GATA3 (catalog no. Hs00231122_m1), JAK1 (catalog no. Hs01026983_m1), IL-4Rα (catalog no. Hs00166237_m1), and STAT6 (catalog no. Hs00598625_m1) were purchased from Applied Biosystems (Foster City, CA). Human IL-4Rα siRNA (catalog nos. s3703, s3704), human JAK1 siRNA (catalog nos. s7646, s7647), human STAT6 siRNA (catalog nos. s13541, s13542), and human GATA3 siRNA (catalog nos. s5600, s5601) were also from Applied Biosystems. A Myc-DDK-tagged ORF clone of a GATA-3 plasmid (catalog no. RC511904) and a Myc-DDK-tagged human NADPH oxidase 1 (Nox1-L) construct (catalog no. RC210426) were obtained from Origene Technologies. RNeasy Mini Kits (catalog no. 74104) and the RNeasy Plus Universal Mini Kit (catalog no. 73404) were from QIAGEN. TaqMan Universal PCR mix (catalog no. 4364340) was from Applied Biosystems. The Dual-Luciferase Reporter Assay System was from Promega Corporation. The FITC BrdU Flow Kit (catalog no. 559619) was from BD Biosciences. The QuikChange II XL Site-Directed Mutagenesis Kit was from Stratagene (catalog no. 200521). The Superoxide Anion Assay Kit was from Sigma-Aldrich (catalog no. CS1000). Protein A/G PLUS-Agarose (catalog no. sc2003), chromatin immunoprecipitation (ChIP) assay lysis buffer (catalog no. sc45000), lysis buffer high salt (catalog no. sc45001), wash buffer (catalog no. sc45002), and elution buffer (catalog no. sc45003) were from Santa Cruz Biotechnology. The NOX1 promoter construct was kindly provided by Dr. Senlin Li (Department of Medicine, University of Texas Health Science Center, San Antonio, TX). The identity of the promoter was confirmed by sequencing.

### Tumor cell culture and *in vitro* growth assay

HT-29 human colon cancer cells were obtained from the American Type Culture Collection (ATCC, Manassas, VA) and grown in McCoy's 5A medium (Lonza, Walkersville, MD) with 10% FBS (Gemini Bio-products, West Sacramento, CA). A stable clone of HT-29 cells that expresses a scrambled NOX1 shRNA (SC cells), and two independent, clonal HT-29 cell lines that express a NOX1 shRNA producing approximately 65-70% (Si6/G6 cells) or > 90% (6A cells) reduction in NOX1 expression have been described previously [16]. WiDr, SW403, NCI-H508, and DLD-1 human colon cancer cell lines were also obtained from ATCC and were propagated in RPMI-1640 medium (GE Healthcare Life Sciences, Logan, UT) with 10% FBS. Tumor cells were cultured in a humidified incubator at 37°C in an atmosphere of 5% CO_2_ in air. Parental HT-29 tumor cells, and the SC, Si6/G6, and 6A clonal variants were seeded into 60 mm tissue culture plates (Sarstedt, Inc., Newton, NC) at a concentration of 1×10^5^ cells/plate in McCoy's 5A medium containing 10% FBS. After one day in culture, cells adherent to the plates were treated with 50 ng/ml of IL-4 or IL-13; cell proliferation was determined by counting each day using a Cellometer Auto T4 Cell Counter (Nexcelom Bioscience, Lawrence, MA). Every sample was measured in triplicate; the data represent a minimum of three independent experiments.

### IL-4Rα inhibition

HT-29 cells were placed in 60 mm tissue culture plates and grown overnight; starved cells that had been grown in medium without serum for 12 h were then pre-treated with IL-4Rα antibody (R & D systems, Minneapolis, MN) for 30 min at concentrations of 1 or 10 ng/ml. IL-4 was then added into the medium for 24 h at a final concentration of 50 ng/ml. Three independent experiments were conducted.

### RNA isolation and real-time RT-PCR

Total cellular RNA was prepared using RNeasy Mini Kits (Qiagen, Valencia, CA). Two micrograms of total RNA was treated with DNAse and reverse transcribed using Superscript II reverse transcriptase (Gibco-BRL, Rockville, MD). 20 μl of cDNA was diluted into 100 μl with water; 2 μl of the cDNA solution was used in every reaction. The integrity of all cDNA preparations was verified by amplifying β-actin as a control gene. Real time RT-PCR was performed on 384-well plates in a 20 μl reaction system containing 1 μl of primer mixture, 7 μl of H_2_O, 10 μl of TaqMan 2 × PCR mixture, and 2 μl of diluted cDNA. The PCR was carried out using default fast thermal cycling conditions (50 °C UNG activation for 2 min, 95°C enzyme activation for 10 min, 40 cycles of 15 s 95°C denaturation, and 1 min for 60°C annealing/extension). Fluorescence was detected with the ABI 7900HT sequence detection system (Applied Biosystems, Foster City, CA). Triplicate determinations were performed for each sample; three separate experiments were performed for each gene of interest.

### Detection of human NOX1 mRNA

PCR primers (designed for both the NOX1-L and NOX1-S transcripts or designed for the NOX1-L transcript alone) were used to detect human NOX1 mRNA. NOX1-L/NOX1-S: 5′-GGGCTTTCGAACAACAATAT-3′ and 5′-CGAGGGCCACATAAGAAAA-3′. NOX1-L: 5′-TGGAGGAATTAGGCAAAGTG-3′ and 5′-CAAAGGAGGTTTTCTGTTTCAG-3′. The NOX1-L/NOX1-S primer set was designed to produce 502- or 355-base pair amplicons depending upon the presence (NOX1-L) or absence (NOX1-S) of exon 11 in NOX1. The NOX1-L primer set was designed to amplify a 141-base pair amplicon from NOX1-L transcripts. The Myc-DDK-tagged human NOX1-L construct (Origene, Rockville, MD) was used as a positive control in our experiments. 2 μl of the cDNA solution was used in standard PCR conditions including 95°C initial denaturation for 30 sec; every cycle had a 30 sec 95°C denaturation, 30 sec 55°C annealing, and 68°C extension; the final extension was performed at 68°C for 5 min. All cell line samples underwent 30 cycles of amplification. Three separate experiments were performed.

### Western analysis

Tumor cells were washed with 1 x PBS (Lonza, Walkersville, MD) three times. Whole cell lysates were prepared in 1 x RIPA lysis buffer (Millipore/Upstate Biotechnology, Temecula, CA) supplemented with 1 tablet of complete mini protease inhibitor and 1 tablet of PhosStop phosphatase inhibitor mix, both from Roche (Indianapolis, IN). After quantitation of protein levels using the BCA protein assay (Thermo Scientific, Rockford, IL), equal amounts (50-100 μg) of protein were loaded on 4-20% TRIS/glycine gels (Invitrogen, Carlsbad, CA) and transferred to nitrocellulose membranes using the iBlot™ Dry Blotting System (Invitrogen, Carlsbad, CA). Membranes were blocked with 5% non-fat dry milk in TBST (TBS, Quality Biologicals, Gaithersburg, MD; containing 0.1% Tween 20) and incubated with a 1:500-5000 dilution of a primary antibody overnight at 4°C. The membranes were then washed with TBST and incubated with the appropriate horseradish peroxidase-conjugated secondary antibody (Santa Cruz Biotechnology, Inc. Santa Cruz, CA) using 1:3000 dilutions for 1 h at room temperature. Specific antibody binding was detected using a chemiluminescence detection system (GE/Amersham Biosciences, UK). Subcellular fractionation of tumor cells was performed as previously described [73].

### Superoxide anion assay

Superoxide anion [O_2_·^−^] was detected using a luminol-based Superoxide Anion Assay Kit (Sigma). Human colon cancer cell lines, including HT-29 (parental and clonal variants as described above), WiDr, or DLD-1 cells were treated with PBS or IL-4/IL-13 (50ng/ml) for 24 h. Following cytokine or PBS exposure, tumor cells were resuspended in 1 ml of fresh medium and counted using the Cellometer. Collected cells were resuspended in assay medium at a concentration of 1 × 10^6^ cells/100 μl. Following the manufacturer's instructions, each well of a 96-well plate contained a final volume of 200 μl; the experimental wells (for cytokine or PBS exposed cells) had 89 μl of assay buffer, 5 μl of luminol solution, 5 μl of enhancer solution, and 1 μl of a 40 μM working stock of phorbol 12-myristate 13-acetate (PMA); control wells were identical except that they lacked PMA and contained 90 μl of assay buffer. Both cytokine-exposed and control cells were also tested in the presence of superoxide dismutase (1 μl of a 4 units/μl stock solution). The reaction components were added to the 96-well plates and mixed by pipetting. The reaction was started by adding 100 μl of the cell suspension in assay medium to each well. Luminescence was detected at 37°C using a GloMax^®^ Microplate Luminometer (Promega BioSciences, San Luis Obispo, CA) with measurements taken every 2 min during a 2 h period of observation. Every sample was measured in triplicate; the data represent a minimum of three independent experiments.

### Determination of intracellular reactive oxygen production by flow cytometry

HT-29 cells were treated with 50 ng/ml of IL-4 for 96 h. DPI (200 nM) was added to the tissue culture medium 2 h before the determination of ROS production. Tumor cells were trypsinized and counted; 1 × 10^6^ cells were resuspended in 1 ml of PBS containing 5 μM of the redox-sensitive dye CM-H_2_-DCFDA (Invitrogen, catalog number C6827), and incubated in the dark for 30 min at 37°C. For short term IL-4 exposure (5 min), 50 ng/ml of IL-4 was added to the cells, and the mixture was incubated in the dark for an additional 5 min. Fluorescence was quickly recorded on the FL-1 channel of a FACS Aria flow cytometer (BD Bioscience) and analyzed using FlowJo^®^ Software.

### Chromatin immunoprecipitation

Chromatin immunoprecipitation [ChIP] analysis was performed for HT-29 cells cultured for 24 h with or without IL-4 treatment; following exposure to IL-4 or vehicle, tumor cells were cross-linked for 10 min with formaldehyde at room temperature; the cross-linking was stopped by adding glycine at a final concentration of 125 mM. Cells were washed and lysed to collect nuclei. Nuclei were resuspended in high salt buffer and sonicated on ice three times (15 s each) using a Sonic Dismembrator Model 100 sonicator (Fisher Scientific) at level 3 to shear chromatin at an average length of ≈ 600 bp. Samples were washed with a protein A/G agarose slurry. The supernatant fraction was collected after centrifugation at 12,000 x *g* for 10 min. Twenty percent of the total supernatant was used as the input control. The remaining eighty percent of the supernatant was used for immunoprecipitation; 5 μg of antibody (either anti-GATA3 or isotype-matched IgG) was incubated with the samples at 4°C overnight. DNA was purified and used in quantitative PCR reactions (SYBR Green qPCR SuperMix, Invitrogen); the primers employed were as follows: 5′CCTCCCTACTTCTCCTGAAGTAATC-3′ and 5′- GAGAACCACAAGGGTTTTACCTGT-3′.

### Immunoprecipitation

Whole cell lysates were prepared in 1 x RIPA lysis buffer (Millipore/Upstate Biotechnology, Temecula, CA) in the presence of 1 tablet of Complete Mini protease inhibitor and 1 tablet of PhosStop phosphatase inhibitor mix, both from Roche (Indianapolis, IN). Lysates were centrifuged at 4°C for 10 min at 12,000 rpm in an Eppendorf microcentrifuge to remove cellular debris. After pre-washing with protein A/G agarose, samples were then immunoprecipitated with 2 μg of anti-GATA3 antibody using protein A/G agarose slurry in the presence of protease inhibitors. The same amount of IgG was used as a negative control. Western analysis was performed using anti-GATA3 and anti-phosphoserine antibodies.

### FITC BrdU flow cytometry

BrdU incorporation into DNA was detected using an FITC BrdU Flow Kit (BD Biosciences, San Jose, CA). Colon cancer cells were labeled by adding BrdU solution into culture medium to produce a final BrdU concentration of 10 μM. The labeled cells were incubated for 1 h at 37°C, collected by trypsinization, centrifuged, and fixed in BD Cytofix/Cytoperm Buffer. Cells were then permeabilized with BD Cytoperm Permeabilization Buffer Plus, and fixed again with BD Cytofix/Cytoperm Buffer. BrdU epitopes were exposed by re-suspending the tumor cells in DNase (30 μg DNase/10^6^ cells) at 37 °C for 1 h. BrdU was stained with a fluorochrome-conjugated anti-BrdU antibody (1:50 dilution, room temperature for 20 min). Total DNA was stained with 20 μl of a 7-aminoactinomycin D [7-AAD] solution (provided with the Kit) using a 5 min incubation. Tumor cells were then resuspended in 1 ml PBS. Stained cells were subsequently analyzed using a FACS Calibur Flow Cytometer (Becton Dickinson, San Jose, CA) equipped with a 488-nm laser capable of detecting both 7-AAD and FITC.

### Protein tyrosine phosphatase activity

Protein tyrosine phosphatase [PTP] activity was detected using PTP Assay Kit 1 (EMD Millipore, catalog number 17-125). Cells (1 × 10^5^) were placed in 60 mm plates; 50 ng/ml of IL-4 was added to culture medium for 24 or 96 h of treatment. Cells were scraped from the dishes with 0.3 ml of phosphatase extraction buffer containing 20 mM imidazole-HCl, 2 mM EDTA, 2mM EGTA, pH 7.0 that included 10 μg/ml each of aprotinin, leupeptin, antipain, soybean trypsin inhibitor, 1 mM benzamidine, and 1 mM PMSF. Cells were centrifuged at 2000 × g for 5 min. Supernatants were used for phosphatase activity assays; 5 μl of 1 mM phosphopeptide was added to 250 ng protein for each sample. Enzyme reactions were performed in a final volume of 25 μl in 96-well microtiter plates incubated at room temperature for 15 min. Color was developed by addition of 100 μl of Malachite Green Solution into each sample for 15 min at room temperature. Absorbance was measured at a wavelength of 620 nm in a microtiter plate reader. Phosphate release was determined by comparing the measured absorbance to a standard curve over a range of 0-2000 pmoles. Each value in the figure represents the mean ± SD of triplicate experiments.

### Construction of point mutations

Point mutations in the human NOX1 promoter and in a human GATA3 construct were obtained using the Quikchange XL mutagenesis kit (Stratagene, La Jolla, CA) following the manufacturer's specifications. For NOX1 promoter construct mutations, the following primer sets were used:

Primer1F

(5′-AGGGGAAGAAGGAAGATGTGATCA GGGAGGGAAATACAAAGAGCTTTAAGATACTG-3′)

Primer 1R

(5′-GATCACATCTTCCTT CTTCCCCTCCTCTACCCCACCGGATGTAATCA-3′)

Primer2F

(5′-TTAGGTCATGTTAAGGAGATGATGAGAGAG AATATTTTCATCCAAGAATGTTGCTATTTC-3′)

Primer2R

(5′-CTCATCATCTCCTTAACATGAC CTAATGTGAAGCATTGCCTTCCTAGATAAAAGA-3′)

Primer 3F

(5′-GTTCTCATAGGAAGGGCT GGTCTATCTAAGCTGGAAGCACAGTTCTGTCC-3′)

Primer 3R

(5′-ACCAGCCCTTCCTA TGAGAACCACAAGGGTTTTACCTGTGGGGATT-3′)

Primer 4F

(5′-CTATCTAAGCTGGAAGC ACAGTTCTGTCCAGAGAAGCTCGAGATCTGCG-3′)

Primer 4R

(5′-AGAACTGTGCTTCCAGCT TAGATAGACCAGCCCTTCCTATGAGAACCACA-3′)

For the GATA3 S308A point mutant:

(F:5′-CAAGCCCAAGCGAAGGCTGGCGgCAGCAAGGAGAGCAGGG-3′;R:5′

CCCTGCTCT CCTTGCTGCCGcCAGCCTTCGCTTGGGCTTG-3′).

### Transient transfection and luciferase assay

Tumor cells were collected with trypsin and counted; 1×10^6^ cells were resuspended in 100 μl of transfection reagent solution (Lonza Amaxa transfection kit, Kit R, Program W-017 for HT-29 cells; Kit T, Program B-024 for WiDr cells) and then transferred to a new tube; for each transfection, 2 μg of plasmid DNA was added and mixed. Cells were electroporated using the Amaxa Nucleofector Device (Lonza, ME). Following transfection, cells were transferred from 100 μl aluminum electrode cuvettes into 60 mm tissue culture plates, and propagated at 37°C overnight in normal tissue culture medium (McCoy's 5A medium with 10% FBS for HT-29 cells; RPMI-1640 medium with 10% FBS for WiDr cells). Fresh tissue culture medium was then exchanged following overnight culture. Firefly/Renilla luciferase were detected on the second day (24 h) and third day (48 h) following transfection using the Dual-Luciferase Reporter Assay System (Promega BioSciences, San Luis Obispo, CA) according to the manufacturer's recommendations.

### NOX1 and IL-4Rα mRNA expression levels in human colon cancer samples

NOX1 and IL-4Rα gene expression determinations from human colon cancers and adjacent normal tissues were accessed from the publicly available portion of the Oncomine^™^ website (NOX1: 206418_at; IL-4R:203233_at). The data for these NOX1 expression levels in human colon cancers had been deposited in the NCBI Gene Expression Omnibus (Series GSE20916). Fresh, pathologically-confirmed primary human colon cancer tissues and adjacent normal tissues were obtained from surgical specimens by the National Cancer Institute-supported Cooperative Human Tissue Network (Eastern, Western, Mid-Western, and Mid-Atlantic Divisions) in compliance with the office of human subjects research at the National Institutes of Health. Specimens were selected without regard to age, race/ancestry, or sex and were acquired from patients who had not received chemotherapy or radiation therapy prior to surgical intervention. Tumors were preserved by snap-freezing in liquid nitrogen within 60 minutes of surgery. Tissues ranging in size from 300 to 2200 mg were homogenized on ice; RNA was isolated utilizing the RNeasy Plus Universal Mini Kits (Qiagen, Valencia, CA, catalog number 73404) according to the manufacturer's protocol. cDNA synthesis and RT-PCR were performed as described above.

### Statistical analysis

Results are expressed as the mean ± standard deviation from at least triplicate experiments. Statistical differences between mean values of control and treated samples were assessed using Student's *t* test; *P* < 0.05 was considered statistically significant. Significance levels were designated as * = *P* < 0.05, ** = *P* < 0.01, and *** = *P* < 0.001 throughout.

### Abbreviations

NOX, NADPH oxidase; DUOX, dual oxidase; ROS, reactive oxygen species; IFN-γ, interferon-γ; TNF-α, tumor necrosis factor-α; IL-4, interleukin-4; DPI, diphenylene iodonium; DTI, 2-di-thienyl-iodonium; IL-13, interleukin-13; MAPK, mitogen activated protein kinase; T_H_2, T helper type 2; IL-4Rα, Type II IL-4 receptor; PMA, phorbol myristate acetate; SOD, superoxide dismutase; siRNA, small interfering RNA; H_2_-DCFDA, 2′,7′-dichlorodihydrofluorescein diacetate; 7-AAD, 7-aminoactinomycin D; CHIP, chromatin immunoprecipitation; PTP, protein tyrosine phosphatase

## SUPPLEMENTARY MATERIALS FIGURES AND TABLES


