# Supplementary material for: Interleukin-4 and interleukin-13 increase NADPH oxidase 1-related proliferation of human colon cancer cells
Source: Oncotarget. 2017 Apr 27;8(24):38113–35. doi: 10.18632/oncotarget.17494 (PMC5503519; doi:10.18632/oncotarget.17494)
Supplement: Supplementary file 1 [file oncotarget-08-38113-s001.pdf]

Interleukin-4 and interleukin-13 increase NADPH oxidase 1-related proliferation of human colon cancer cells

Supplementary Material

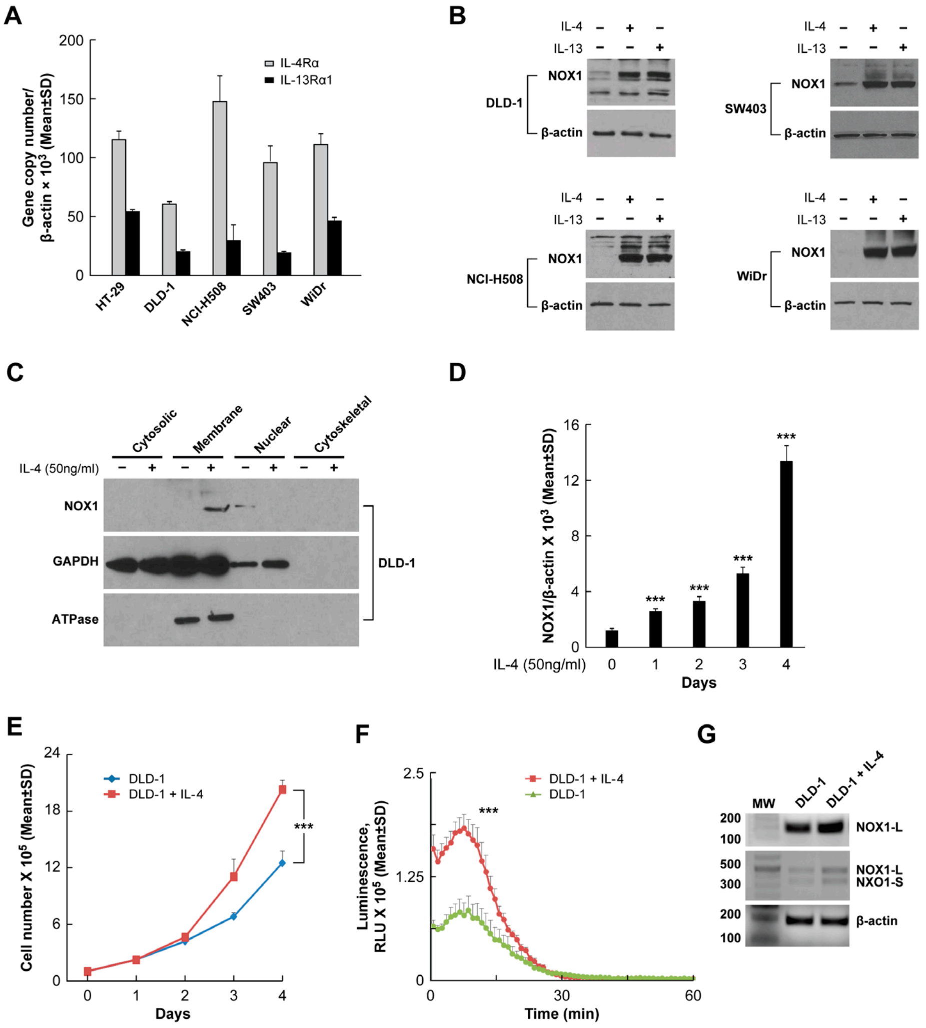

Supplementary Figure S1: Effects of treatment with IL-4 and IL-13 on NOX1 expression and function in human colon cancer cell lines. (A) 5 human colon cancer cell lines (HT-29, DLD-1, NCI-H508, SW403, and WiDr) display high levels of IL-4Rα and IL-13Rα1 expression when analyzed by quantitative RT-PCR with β-actin serving as the internal control. (B) NOX1 expression can be induced by IL-4 or IL-13 in colon cancer cell lines in addition to the HT-29 line, that express IL-4Rα and IL-13Rα1. Western analysis was performed on DLD-1, NCI-H508, SW403, and WiDr cells that were exposed to 50 ng/ml IL-4 or IL-13 for 24 h with β-actin serving as an internal control. (C) IL-4-induced NOX1 protein localizes to the plasma membrane in DLD-1 cells treated with IL-4 (50 ng/ml) for 24 h. Western analysis was performed on different cellular fractions (cytosolic, membrane, nuclear and cytoskeletal) to determine NOX1 localization. GAPDH and ATPase were used as cytosolic and membrane markers, respectively. (D) IL-4 treatment increases NOX1 expression in DLD-1 cells during 4 days of continuous IL-4 exposure; NOX1 levels were determined by quantitative RT-PCR with β-actin serving as the internal control. (E) IL-4 promotes DLD-1 cell proliferation. Cell numbers were counted every day in the presence or absence of IL-4 for 4 days. After completion of IL-4 treatment, compared with the corresponding untreated control, DLD-1 cells demonstrated a significant increase in cell number. (F) IL-4 exposure for 24 h enhances superoxide production in DLD-1 cells; superoxide production was detected using Superoxide Anion Assay Kit as described in the Materials and Methods. (G) IL-4 induces the expression of full-length *NOX1* in DLD-1 cells following treatment for 24 h. PCR and subsequent DNA gel analysis were performed to detect *NOX1-L* and *NOX1-S* in IL-4-treated DLD-1 cells; β-actin served as the internal control. Data are shown as the mean ± SD of at least three experiments. *** = *P* <0.001.

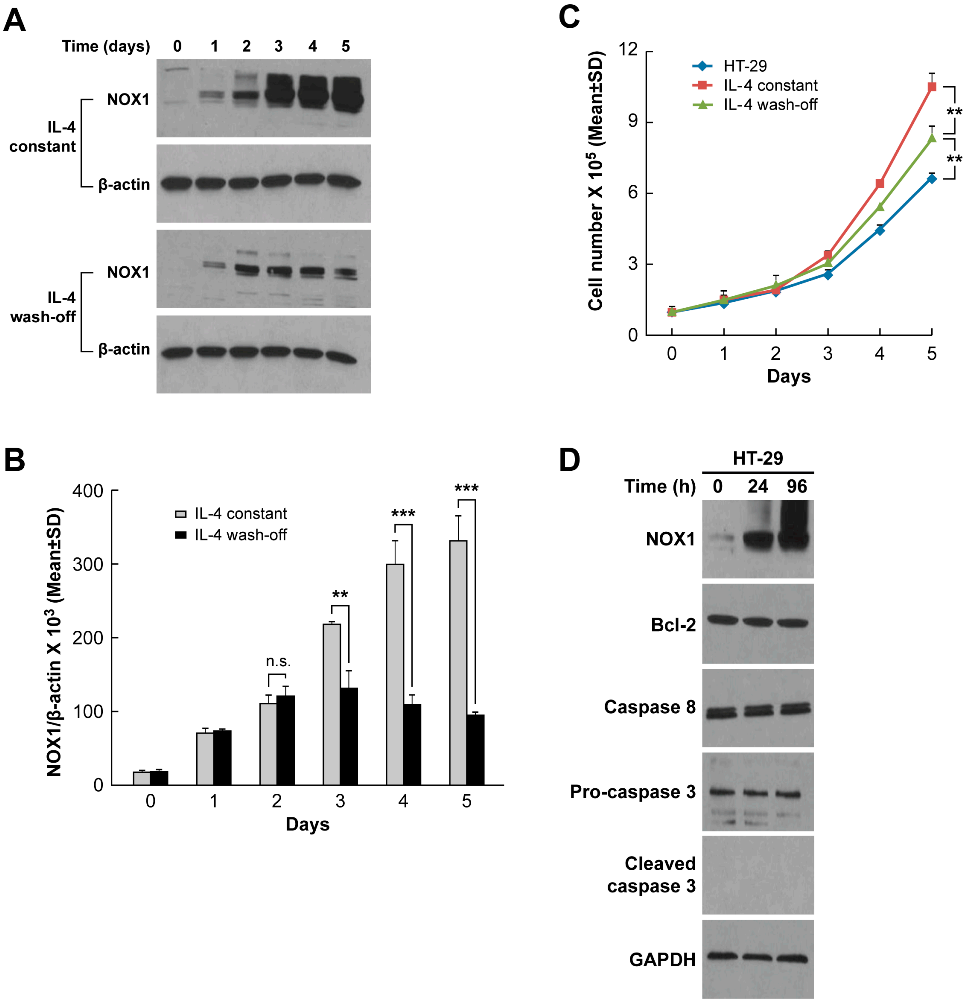

Supplementary Figure S2: Kinetics of NOX1 expression in HT-29 cells following IL-4 (50 ng/ml) exposure for 120 h (constant) or after 24 h IL-4 exposure with washing, removal of the cytokine, and replacement with fresh media (wash-off). (A) Western analysis of HT-29 cells for NOX1 expression from the two treatment groups was performed to evaluate the kinetics of NOX1 expression and decay; β-actin served as the loading control. (B) NOX1 mRNA expression levels analyzed by quantitative RT-PCR for the same two treatment groups; β-actin served as the internal control. (C) Cell proliferation for parental HT-29 cell controls was compared to the growth of HT-29 cells exposed to IL-4 (50 ng/ml) for 120 h, or to IL-4 (50 ng/ml) for 24 h, followed by replacement with fresh media without IL-4. Data represent the mean ± SD of three independent experiments. (D) Western analysis was performed on HT-29 cells treated with IL-4 (50ng/ml) at different time points (0, 24, and 96 h). The relationship between NOX1 expression and the apoptotic cascade was probed with specific antibodies against Bcl-2, caspase 8 and caspase 3; GAPDH served as the loading control. ** = $P < 0.01$; *** = $P < 0.001$; n.s. = not significant.

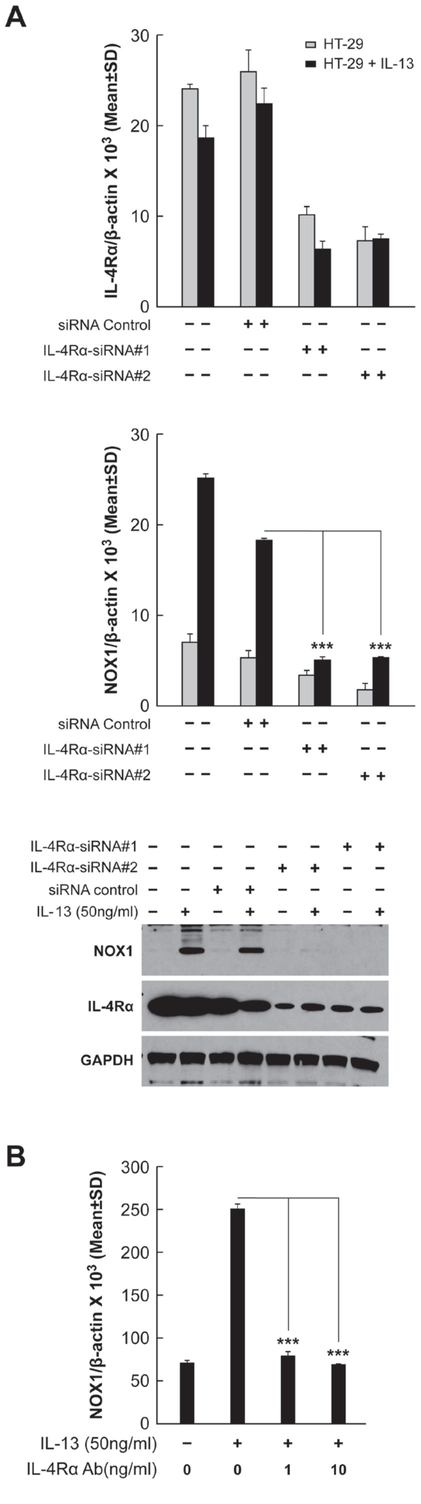

Supplementary Figure S3: IL-13 upregulates NOX1 expression through the IL-4R/JAK1/STAT6 signaling pathway. (A) IL-4Rα knockdown blocks IL-13-related NOX1 expression at both the mRNA (upper and middle panels) and protein level (lower panel). HT-29 cells were transiently transfected with either of the two different IL-4Rα- specific siRNAs and treated with IL-13 (50 ng/ml) 24 h later. Cells were harvested 24 h following IL-13 treatment and examined by quantitative RT-PCR and Western analysis. β-actin and GAPDH served as the internal controls. (B) Effect of the concurrent presence of an IL-4Rα antibody on the stimulation of NOX1 expression by IL-13 in HT-29 cells was examined in the absence of FBS and measured by quantitative RT-PCR. β-actin served as the loading control. Data represent the mean ± SD of three experiments. *** = $P < 0.001$.

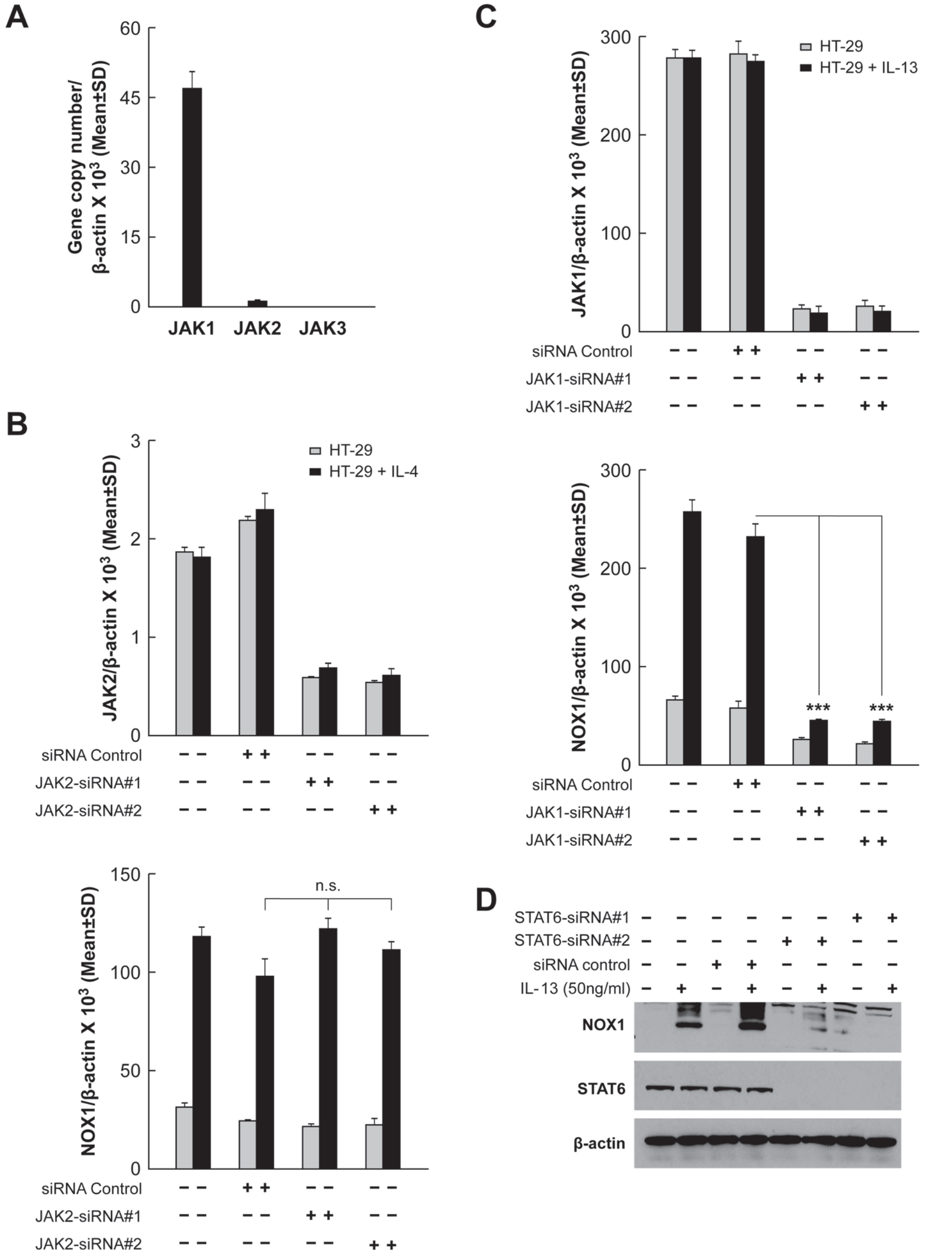

Supplementary Figure S4: Janus Kinase family member expression and IL-4-induced NOX1 expression. (A) Expression levels of three different JAK family members were detected using quantitative RT-PCR. β-actin served as the internal control. (B) IL-4-induced NOX1 expression is JAK2-independent. HT-29 cells were transiently transfected with either of two different JAK2-specific siRNAs and treated with IL-4 (50 ng/ml) 24 h later. Cells were harvested at 24h post-treatment and examined with quantitative RT-PCR. (C) JAK1 knockdown blocks IL-13-stimulated NOX1 expression. HT-29 cells were transiently transfected with either of the two different JAK1-specific siRNAs and treated with IL-13 (50 ng/ml) 24 h later. Cells were harvested at 24 h post-treatment and analyzed by quantitative RT-PCR. (D) STAT6 knockdown blocks IL-13-enhanced NOX1 expression. HT-29 cells were transiently transfected with either of the two different STAT6-specific siRNAs and treated with IL-13 24 h later. Cells were harvested at 24 h post-treatment and examined by Western analysis. β-actin served as the internal control. Data represent the mean ± SD of three experiments. n.s. = not significant.

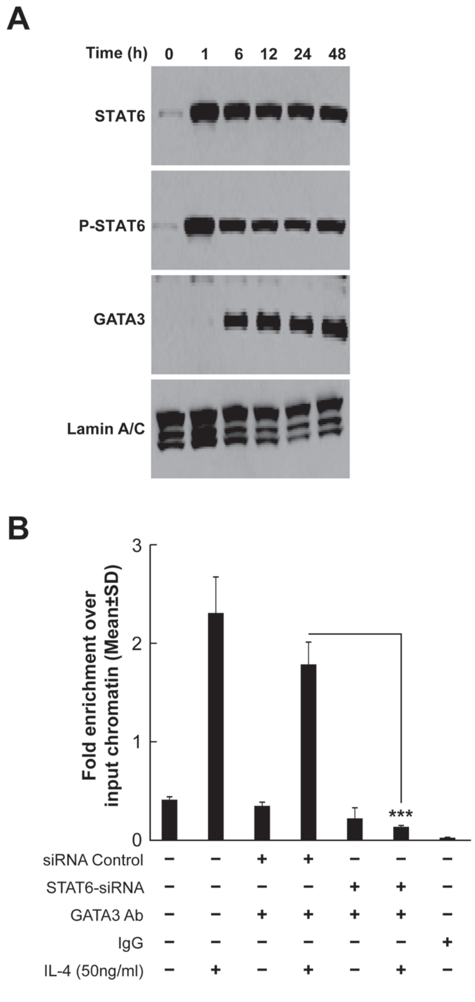

Supplementary Figure S5: GATA3 responds to STAT6 activation following IL-4 treatment. (A) Nuclear fractions from HT-29 cells were prepared following exposure to IL-4 (50 ng/ml) for a variety of time intervals (0, 1, 6, 12, 24 and 48 h), and protein levels of STAT6, phosphorylated STAT6, and GATA3 were examined by Western analysis. Lamin A/C served as the loading control. (B) ChIP assay and subsequent quantitative PCR were performed on HT-29 cells transiently transfected with either siRNA control or STAT6-specific siRNA. IL-4 exposure was for 24 h. Isotype-matched IgG was used as the control. Data represent the mean ± SD of three experiments. *** = $P < 0.001$.

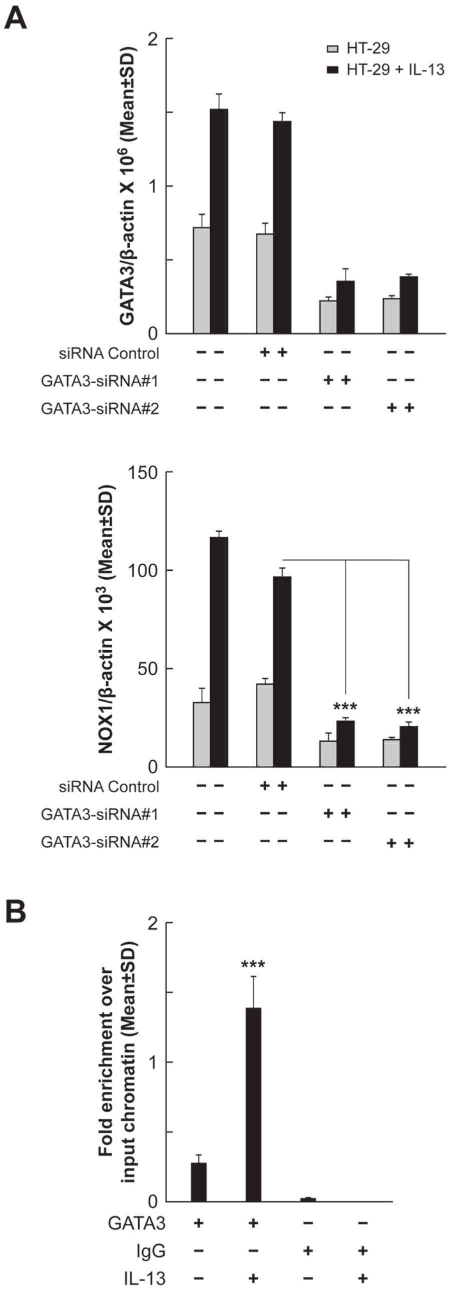

Supplementary Figure S6: GATA3 is involved in IL-13-induced NOX1 expression. (A) HT-29 cells were transiently transfected with either of two different GATA3-specific siRNAs and treated with IL-13 (50 ng/ml) 24 h later. Cells were harvested at 24 h post-treatment and analyzed by quantitative RT-PCR. β-actin served as the internal control. (B) ChIP assay and subsequent quantitative PCR were performed on HT-29 cells exposed to IL-13 (50 ng/ml for 24 h). Isotype-matched IgG was used as the control. Data represent the mean ± SD of three experiments. *** = $P < 0.001$.
